# eIF4B phosphorylation at Ser504 links synaptic activity with protein translation in physiology and pathology

**DOI:** 10.1038/s41598-017-11096-1

**Published:** 2017-09-05

**Authors:** Barbara Bettegazzi, Serena Bellani, Paolo Roncon, Fabrizia Claudia Guarnieri, Alice Bertero, Franca Codazzi, Flavia Valtorta, Michele Simonato, Fabio Grohovaz, Daniele Zacchetti

**Affiliations:** 1 Unit of Cellular Neurophysiology and Unit of Neuropsychopharmacology, Division of Neuroscience, IRCCS San Raffaele Scientific Institute, via Olgettina 60, I-20132 Milano, Italy; 2 Vita-Salute San Raffaele University, via Olgettina 58, I-20132 Milano, Italy; 3 Department of Medical Sciences, University of Ferrara, via Fossato di Mortara 17-19, 44121 Ferrara, Italy

## Abstract

Neuronal physiology requires activity-driven protein translation, a process in which translation initiation factors are key players. We focus on eukaryotic initiation factor 4B (eIF4B), a regulator of protein translation, whose function in neurons is undetermined. We show that neuronal activity affects eIF4B phosphorylation and identify Ser504 as a phosphorylation site regulated by casein kinases and sensitive to the activation of metabotropic glutamate receptors. Ser504 phosphorylation increases eIF4B recruitment to the pre-initiation complex and influences eIF4B localization at synapses. Moreover, Ser504 phosphorylation modulates the translation of protein kinase Mζ. Therefore, by sensing synaptic activity, eIF4B could adjust translation to neuronal needs, promoting adaptive changes in synaptic plasticity. We also show that Ser504 phosphorylation is increased *in vivo* in a rat model of epilepsy during epileptogenesis i.e. when translation drives maladaptive synaptic changes. We propose eIF4B as a mediator between neuronal activity and translation, with relevance in the control of synaptic plasticity.

## Introduction

The eukaryotic translation initiation factor 4B (eIF4B) is part of the eIF4 group of factors, which assist the 43S ribosomal initiation complex in mRNA recruitment and scanning^1, 2^. In particular, eIF4B favors the interaction of the mRNA molecule with the initiation complex, by binding both eIF3 and the 18S ribosomal RNA^3, 4^, and stimulates the helicase activity of eIF4A^5–7^, thereby supporting the translation of mRNAs with highly structured transcript leaders, often coding for proteins involved in vital cellular processes^8^. eIF4B activity is modulated through phosphorylation by various protein kinases^9, 10^. Although several phosphorylation sites have been identified or predicted^11–14^, only Ser422 and Ser406 have been characterized as regulated sites of phosphorylation in proliferating cells^15, 16^. Both sites are targeted by ribosomal S6 kinase (RSK), downstream the mitogen-activated protein kinase (MAPK) pathway, and by S6 kinase 1 (S6K1), activated through the phosphoinositide 3-kinase (PI3K)/mammalian target of rapamycin (mTOR) axis^15–17^. Phosphorylation of Ser422 modulates the interaction of eIF4B with eIF3^4, 17^, whereas phosphorylation of Ser406 is important for optimal translational activity of eIF4B^16^.

The function of eIF4B has been widely investigated at the molecular level, whereas its characterization at the cellular level is still limited and even contradictory^18^. It has been reported that the state of phosphorylation – and thus the activity – of eIF4B is modulated by several extracellular stimuli^19^ and influences functions as important as cell survival and proliferation^18, 19^. Accordingly, a pro-oncogenic role for eIF4B has been demonstrated in cancer cells, probably because of its ability to positively regulate the translation of pro-survival mRNAs^19^. Despite its ubiquitous expression, very little is known about the role of eIF4B in neurons. These highly specialized cells require a tight control of general and local protein synthesis to rapidly adjust protein composition to the incoming synaptic activity, and thus to sustain plasticity processes^20–22^.

We investigated how perturbations of synaptic activity modulate the expression, phosphorylation, localization and function of eIF4B in mature hippocampal neurons in culture, and validated our results *in vivo* taking advantage of the pilocarpine-induced model of epilepsy.

## Results

### eIF4B expression and phosphorylation in neurons and astrocytes

**Figure 1:**
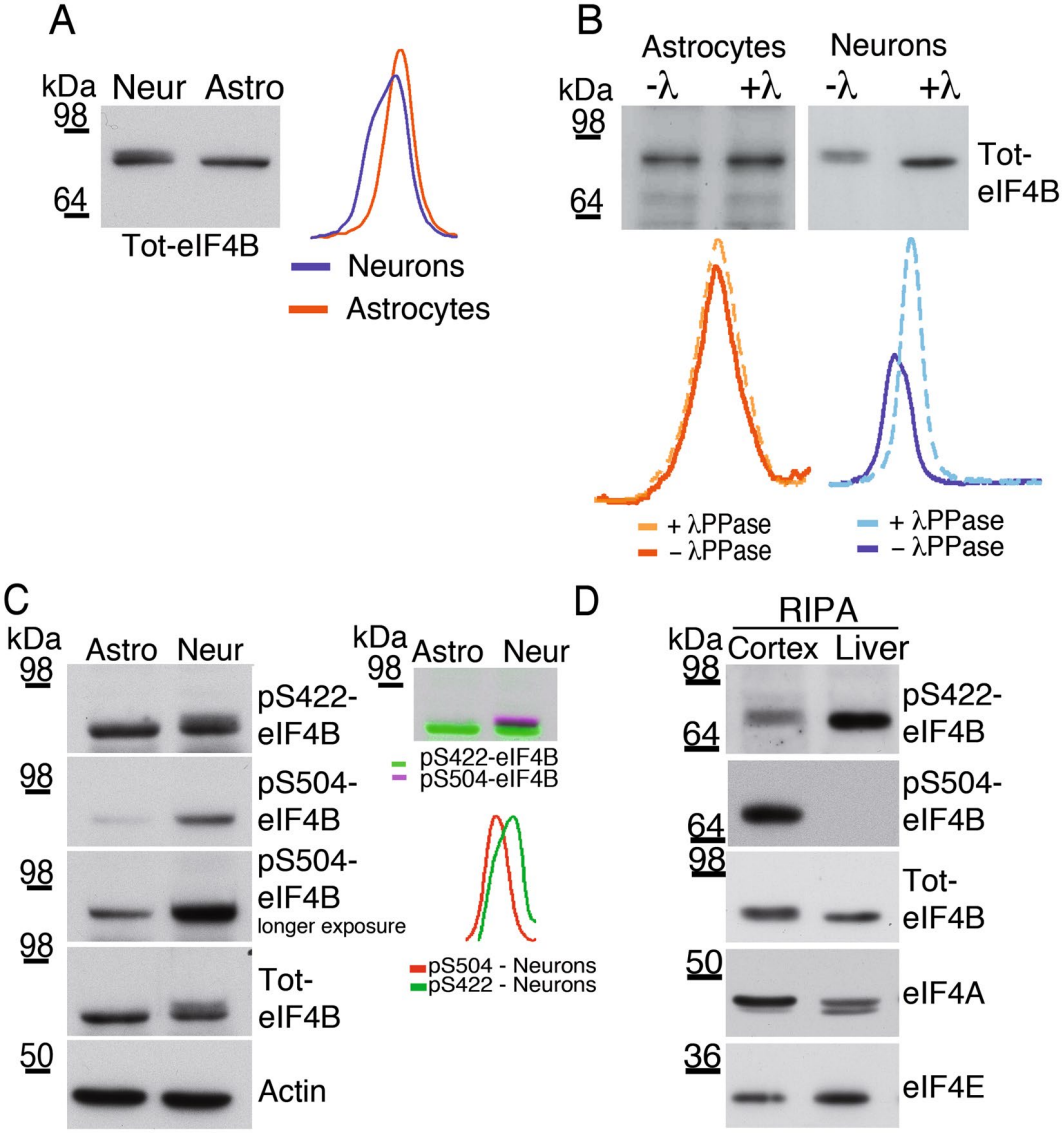
eIF4B phosphorylation patterns in hippocampal neurons and cortical astrocytes. (**A**) Western blot analysis of total cell extracts from neurons or astrocytes using antibodies against either the N-terminal (Nt-eIF4B) sequence of eIF4B. The densitometric profiles show that the N-terminal eIF4B antibody recognizes two bands in neurons but only one band in astrocytes. (**B**) Western blot and densitometric analysis of total cell extracts prepared from neurons or astrocytes, treated with λ-phosphatase (λ), separated by SDS-PAGE and then incubated with the anti-N-terminal eIF4B antibody (Nt-eIF4B). Dephosphorylation of neuron extracts causes a mobility shift of the slowly migrating band of eIF4B; the astrocyte band is unaffected by the treatment. (**C**) On the left, western blot analysis of total cell extracts from neurons or astrocytes: after detection with an antibody against phospho-Ser422 eIF4B (pS422-eIF4B), the membrane was stripped and re-probed with antibodies against phospho-Ser504 eIF4B (pS504-eIF4B) and actin (Actin), as loading control. On the right, the overlay of the pS422-eIF4B and pS504-eIF4B bands shown on the left, together with their densitometric profiles are shown. eIF4B appears as a single band in astrocytes and as a double band in neurons. (**D**) Expression and phosphorylation of eIF4B in tissues. Extracts from adult rat cortex and liver were subjected to SDS–PAGE followed by western blot analysis for phospho-S504 eIF4B (pS504-eIF4B), phosho-S422 eIF4B (pS422-eIF4B), eIF4B (Nt-eIF4B), eIF4A, and eIF4E.

The expression of eIF4B was detected by Western blotting in both rat neuronal and astroglial primary cultures, but with a distinct pattern of electrophoretic migration: in astrocytes a single sharp band of approximately 80 kDa was visible, whereas neurons displayed two close bands of similar MWs (Fig. 1A). Notably, the slowly migrating form of eIF4B disappeared when neuronal lysates were treated with λ-phosphatase (λPPase), indicating that phosphorylation might account for the peculiar migrating behavior in neurons (Fig. 1B). Two-dimensional gel electrophoresis followed by Western blotting further confirmed this observation. In astrocytes, the eIF4B pattern showed multiple spots, likely due to the phosphorylation of different sites (Fig. S1A, upper panel). Neurons displayed an even more complex pattern of eIF4B two-dimensional migration (Fig. S1A, lower panel), suggesting the presence of additional phosphorylation sites in neurons compared to astrocytes.

The electrophoretic shift of eIF4B observed in neurons may be caused by phosphorylation of serine-proline or threonine-proline (SP/TP) sites, which has often been reported to greatly alter the electrophoretic mobility of proteins^23, 24^. Three SP sites (Ser93, Ser459 and Ser504) are present in eIF4B and have been shown to be phosphorylated according to large-scale mass spectrometry analysis of phospho-peptides^12, 25–27^ and data deposited in PhosphoSitePlus^28^. We focused on Ser504, since a phospho-specific antibody was already available for this SP site^29^.

Interestingly, we observed that Ser504 is phosphorylated to a significantly higher degree in neurons with respect to astrocytes (Fig. 1C left panel). As a comparison, the levels of eIF4B phosphorylation on Ser422 were similar in the two cell types. Notably, the phospho-Ser504 signal coincided with the slower migrating band in neurons, while this was not the case for phospho-Ser422 (Fig. 1C, right panel). The result of Western blot analysis of HEK293 cells transfected with Flag-tagged eIF4B constructs harboring different mutations of Ser504 are consistent with the hypothesis that the phosphorylation of this residue is sufficient to alter eIF4B electrophoretic mobility. Indeed, the substitution of Ser504 with either alanine (S504A) or glutamate (S504E, a phosphomimetic mutation) caused higher or lower electrophoretic mobility, respectively (Fig. S1B). The antibody was specific for phosphorylated Ser504 since it recognized neither the two mutant forms of eIF4B in Western blot experiments (unpublished data) nor Flag-tagged eIF4B immuno-precipitated from cell extracts treated with λPPase (Fig. S1C). Furthermore, the phospho-Ser504 immunofluorescence signal in neurons was virtually abolished by alkaline phosphatase treatment (Fig. S2).

The expression and phosphorylation profile of eIF4B was also evaluated in adult rat cerebral cortex and liver samples in order to understand which residues are preferentially phosphorylated *in vivo* in the brain. Strikingly, Ser504 phosphorylation was virtually absent in the liver but high in the cerebral cortex (Fig. 1D). Conversely, Ser422 was highly phosphorylated in liver, and, to a lesser extent, in the cortex.

Based on these considerations, we decided to further characterize the regulation and relevance of Ser504 phosphorylation for eIF4B function in neurons.

### Modulation of eIF4B phosphorylation by changes in neuronal activity: involvement of glutamate receptors and voltage-operated calcium channels

Phosphorylation of eIF4B is reported to play an important role in translation initiation in many cell lines^15–17^. Considering that changes in translation are strictly related to a variety of activity-dependent neuronal processes^30, 31^, we investigated modifications of eIF4B phosphorylation under different conditions of neuronal activity. We focused our attention on the previously uncharacterized pathways of Ser504 phosphorylation and analyzed changes in Ser422 phosphorylation for comparison.

**Figure 2:**
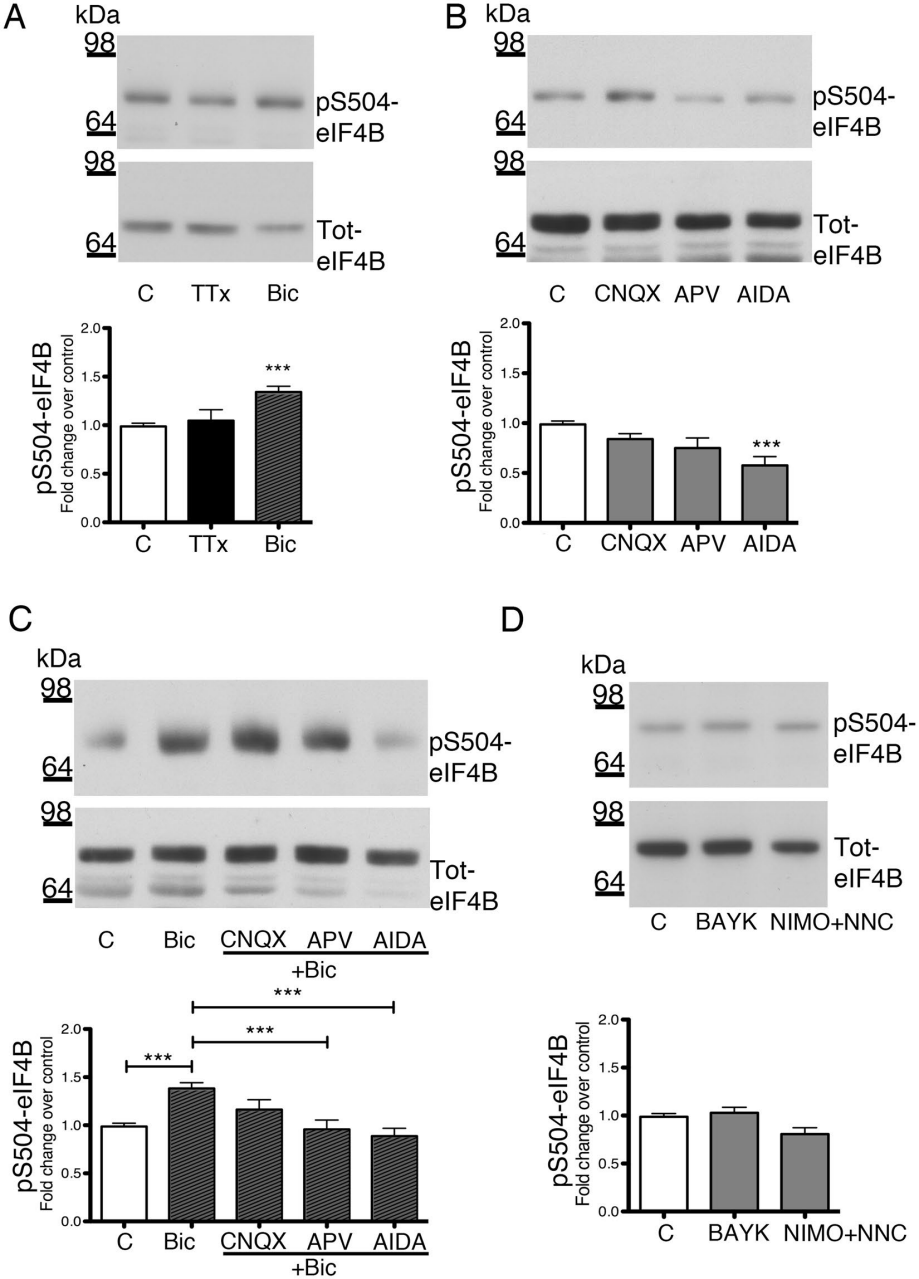
Regulation of Ser504 eIF4B phosphorylation by neuronal activity. (**A**–**D**), Representative western blot (top) and quantification (bottom) of phospho-Ser504 eIF4B (pS504-eIF4B) levels in primary hippocampal neurons exposed to various drugs. (**A**) 1 μM TTx for 60 min (TTx) or 10 μM bicuculline for 30 min (Bic) (n = 4 each group); (**B**) 30-min treatment with the following antagonists of GluRs: 100 μM APV for NMDA-Rs; 20 μM CNQX for AMPA-R/kainate-R; 1 mM AIDA for group I mGluRs (n = 4); (**C**) 30-min treatment with 10 μM bicuculline after a 30-min pre-incubation with GluR antagonists as in B (n = 3 each group); (**D**) 10 μM BAYK8644 (60 min; BAYK) or 10 μM nimodipine +10 μM NNC 55–0396 (60 min; Nimo + NNC) (n = 3 each group). Phosphorylated eIF4B levels are normalized against total eIF4B and shown as fold change over control (untreated neurons). Data are shown as mean ± SEM of at least three independent experiments for each treatment. Statistical significance is calculated using one-way ANOVA followed by Bonferroni post hoc test; ***P < 0.001.

Modulation of the spontaneous network activity was obtained by exposing primary hippocampal neurons to either tetrodotoxin (TTx, 1 μM, 1 h), a sodium channel blocker that inhibits neuronal firing, or bicuculline (10 μM, 30 min), a blocker of the GABA_A_ receptor able to enhance the intrinsic network activity by decreasing inhibitory inputs. Exposure to bicuculline caused an increase in Ser504 phosphorylation, whereas TTx treatment had no effect (Fig. 2A). At variance, Ser422 phosphorylation was increased by bicuculline and decreased by TTx (Fig. S3A).

Since neuronal network activity is mainly sustained by excitatory glutamatergic neurons, we verified the effects exerted by inhibitors of the different glutamate receptors (GluRs) on eIF4B phosphorylation, both at rest and after exposure to 10 μM bicuculline (Fig. 2B and C). More specifically, hippocampal neurons were treated with the following GluR antagonists: CNQX (20 μM) for AMPA (α-amino-3-hydroxy-5-methyl-4-isoxazolepropionic acid) and kainate receptors (AMPA-R/kainate-R); APV (100 μM) for N-methyl-D-aspartate receptors (NMDA-R); and (RS)-1-aminoindan-1,5-dicarboxylic acid (AIDA, 1 mM) for group I metabotropic GluRs (mGluR).

In resting conditions, the blockade of AMPA-R or NMDA-R activity did not affect Ser504 phosphorylation, while the mGluR antagonist AIDA decreased it by almost 50% (Fig. 2B). Stimulation of the network activity by bicuculline caused an increase in Ser504 phosphorylation, which was inhibited by the antagonists of NMDA-R or mGluR, but not of AMPA-R (Fig. 2C). In summary, at rest, Ser504 phosphorylation appears to be mainly controlled by the tonic activation of mGluRs. When network activity is increased, an additional contribution of NMDA-Rs to Ser504 phosphorylation is observed.

In contrast Ser422 phosphorylation appears to be governed mainly by the activity of NMDA-Rs, with an additional component related to AMPA-Rs under increased network activity. In fact, only the NMDA-R antagonist reduced the basal level of Ser422 phosphorylation to values close to those observed after TTx treatment, while both NMDA-R and AMPA-R antagonists were able to inhibit the phosphorylation increase induced by bicuculline (Fig. S3B and C). The antagonist of mGluRs showed no effect on Ser422 phosphorylation.

Since the above results indicate that eIF4B phosphorylation is influenced by synaptic activity, we investigated the role of voltage-operated Ca^2+^ channels (VOCCs), which are known to actively participate in neuronal activity^32^ and to regulate protein phosphorylation^33, 34^. BAYK8644 (10 μM, 60 min) was used to prolong the opening time of the L-type VOCCs^35^, while two different antagonists were employed together to block the activity of most post-synaptic VOCCs: nimodipine (10 μM) for the L-type and NNC55–0396 (10 μM) for the T-type^36–38^. Modulation of VOCC activity by either BAYK8644 or the mix of VOCC blockers had virtually no effects on the levels of Ser504 phosphorylation (Fig. 2D) while BAYK8644 and the mix of VOCC blockers increased and reduced, respectively, eIF4B Ser422 phosphorylation levels (Fig. S3D).

Altogether, these findings provide evidence that Ser504 and Ser422 phosphorylation are differentially regulated by neuronal activity.

### Role of intracellular kinases involved in eIF4B phosphorylation

Considering that RSK and S6K1 protein kinases are already known to phosphorylate eIF4B at Ser422, we addressed the possible role of these kinases in modulating also eIF4B Ser504 phosphorylation in neurons by using specific inhibitors of their upstream activators. Hippocampal neurons, either at rest or activated by 10 μM bicuculline, were pre-treated with the following inhibitors: U0126 (15 μM) for the MAPK kinase pathway; rapamycin (20 nM) for mTOR; and LY294002 (25 μM) for PI3K.

**Figure 3:**
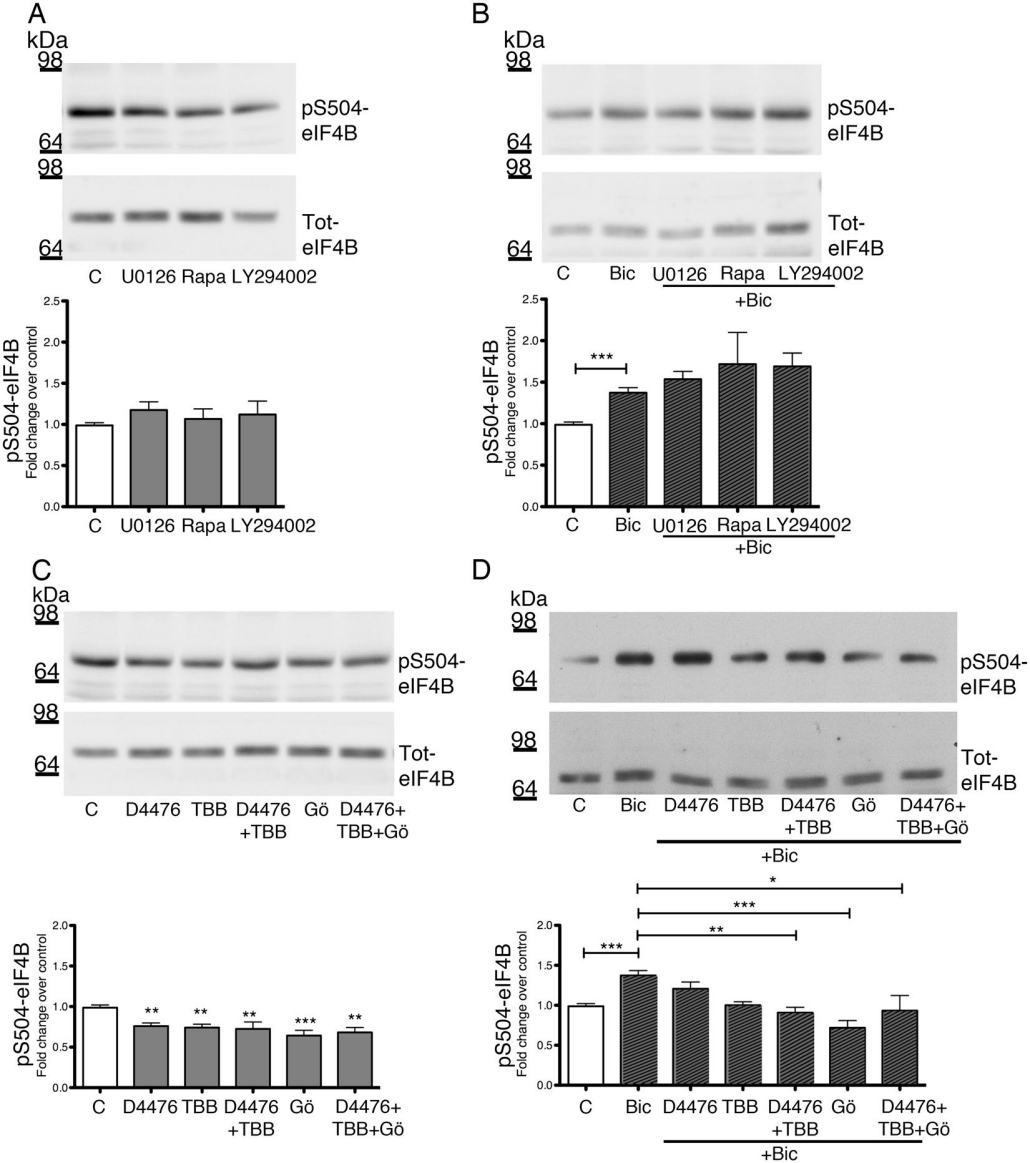
Identification of the protein kinases involved in the Ser504 phosphorylation of eIF4B in neurons. (**A**–**D**), Representative western blot and quantification of phospho-Ser504 eIF4B (pS504-eIF4B) levels in primary hippocampal neurons treated for 3 h with 15 μM U0126, 20 nM rapamycin (Rapa) or 25 μM LY294002 (**A**,**B**) or pre-treated for 2 h with two CK inhibitors (10 μM D4476, 25 μM TBB) or a cPKC inhibitor (2 μM Gö6976; Gö), alone or in combination (**C**,**D**) and analyzed at rest (**A**,**C**) or after treatment with 10 μM bicuculline (Bic) for 30 min (**B**,**D**). Phosphorylated eIF4B levels are normalized against total eIF4B and shown as fold change over control (untreated neurons). Data are shown as mean ± SEM of at least three independent experiments for each treatment ((**A**,**B**) n = 3; (**C**,**D**) n = 4). Statistical significance is calculated using one-way ANOVA followed by Bonferroni post hoc test; *P < 0.05, **P < 0.01, ***P < 0.001.

In accordance with previous studies in non-neuronal cells, basal Ser422 phosphorylation was significantly affected by both rapamycin and LY294002, while U0126 had no effect (Fig. S4A). However, only U0126 was able to prevent the increase in eIF4B Ser422 phosphorylation induced by bicuculline (Fig. S4B), probably due to MAPK activation upon stimulation of neuronal activity. On the other hand, Ser504 phosphorylation was not affected by blockade of MAPK, mTOR or PI3K pathways, both at rest and after bicuculline treatment, indicating that other kinases are responsible for eIF4B phosphorylation at this residue (Fig. 3A and B).

In order to identify the kinases mediating phosphorylation of eIF4B at Ser504, we searched for kinase-specific phosphorylation motifs encompassing this residue with the bioinformatics tools PhosphoMotif Finder^39^ and Group-based Prediction System (GPS 2.1 software)^40^. We found that Ser504 is located in putative consensus sequences for the following kinases: MAPKs, cyclin-dependent kinase 5 (CDK5), casein kinase (CK) 1 and 2, and conventional isoforms of PKC (cPKCs). Having already excluded a direct contribution of MAPKs, we used specific inhibitors for the other kinases: 20 uM roscovitine for CDK5^41^; 10 uM D4476 for CK1^42^; 25 uM TBB for CK2^43^ and 2 uM Gö6976 for cPKCs^44^. While roscovitine was ineffective in modulating eIF4B phosphorylation (not shown), D4476, TBB and Gö6976 were able to significantly reduce the basal levels of Ser504 phosphorylation (Fig. 3C). The increase in Ser504 phosphorylation elicited by bicuculline was also reduced by the two CK inhibitors, alone or in combination, and to a larger extent by the cPKC inhibitor; no synergistic effect was observed when cPKC and CK inhibitors were added in combination (Fig. 3D). Interestingly, while the PKC inhibitor exerted its effect also on Ser422, suggesting a general effect of PKC on eIF4B phosphorylation, CK inhibitors did not decrease Ser422 phosphorylation, either at rest or after exposure to bicuculline (Fig. S4C and D), indicating a specific role of CKs on Ser504 phosphorylation.

### Subcellular localization of eIF4B in neurons

**Figure 4:**
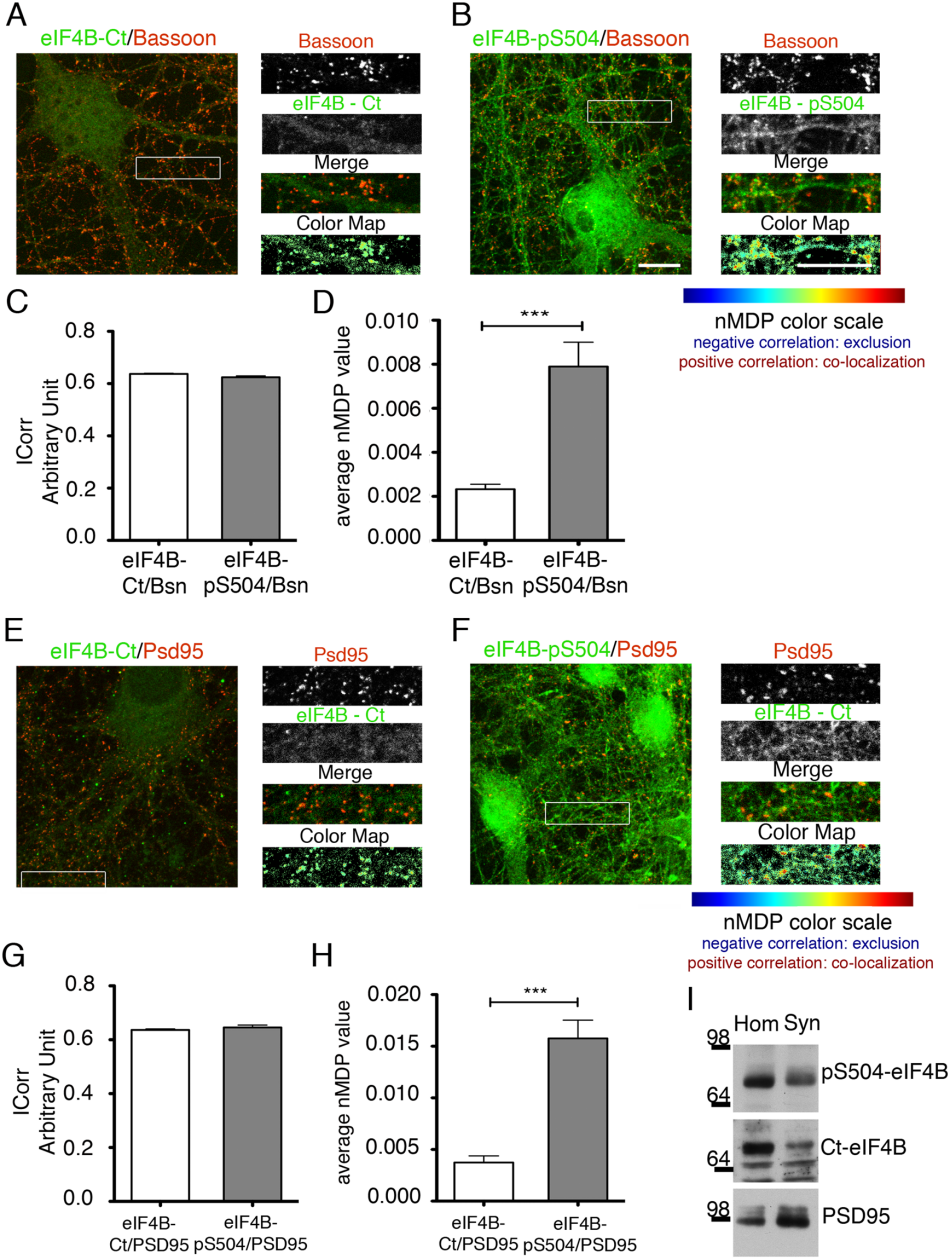
Subcellular localization of eIF4B in neurons. (**A**,**B**,**E**,**F**) Immunofluorescence with antibodies against total (eIF4B-Ct, green; **A**,**E**) or Ser504 phosphorylated eIF4B (eIF4B-pS504, green; **B**,**F**) and the synaptic proteins Bassoon (red; **A**,**B**) or PSD-95 (red; **E**,**F**). Total eIF4B shows a diffuse distribution consistent with cytosolic localization, while eIF4B with Ser504 phosphorylation displays a punctate staining localized in neurites. The Color map pictures (right column) show the images of the normalized mean deviation product (nMDP) between two channels, wherein hot colors represent co-localization, and cold colors indicate mutual exclusion. (**C**,**D**,**G**,**H**) Quantification of the co-localization of eIF4B with Bassoon is expressed by the ICorr score (**C**,**G**) and by the average nMDP score (**D**,**H**). Data are expressed as mean values ± SEM; n = 30 pictures from three independent experiments. Statistical significance is determined with a Mann-Whitney U test; ***P < 0.0001. (**I**) Representative western blot of phospho-Ser504 eIF4B (pS504-eIF4B) or total eIF4B in crude brain homogenate (Hom) and synaptosomes (Syn) from adult rat cortex (n = 3). The Syn fraction is enriched in both PSD95 and Ser504 phosphorylated eIF4B. Bar in (**A**,**B**,**E**,**F**): 10 μm.

Recent data have shown that Ser406-phosphorylated eIF4B partially localizes at synapto-dendritic domains^45^. We evaluated whether also Ser504-phosphorylated eIF4B displays a specific subcellular enrichment. Total eIF4B appeared localized in soma and neurites, with a uniform signal consistent with a cytosolic localization (Fig. 4A). Ser504-phosphorylated eIF4B displayed a more punctate localization, reminiscent of synaptic bouton staining (Fig. 4B). The co-localization between Bassoon, a presynaptic marker, and either total eIF4B or Ser504-phosphorylated eIF4B was investigated by confocal microscopy and analyzed by the ImageJ Colormap software^46^ (see Materials and Methods). Co-localization was quantified in terms of index of correlation (ICorr), a measure of the fraction of positively correlated pixels for the two signals. Similar ICorr values were obtained when the signal for Bassoon was compared with that of total or Ser504-phosphorylated eIF4B (Fig. 4C), demonstrating that a fraction of the eIF4B protein is present in synaptic puncta. However, the average normalized mean deviation product (nMDP, a measure of the relative intensity of the two signals within individual pixels) was higher for Ser504-phosphorylated than for total eIF4B (Fig. 4D), indicating a preferential localization of the phosphorylated protein at synaptic microdomains. Similar results were obtained for the co-localization of either total or Ser504-phosphorylated eIF4B with a postsynaptic marker, the postsynaptic density protein of 95 kDa (PSD95; Fig. 4E–H). Consistently, Ser504-phosphorylated eIF4B was enriched in the synaptosomal fraction of rat brain homogenates (Fig. 4I).

**Figure 5:**
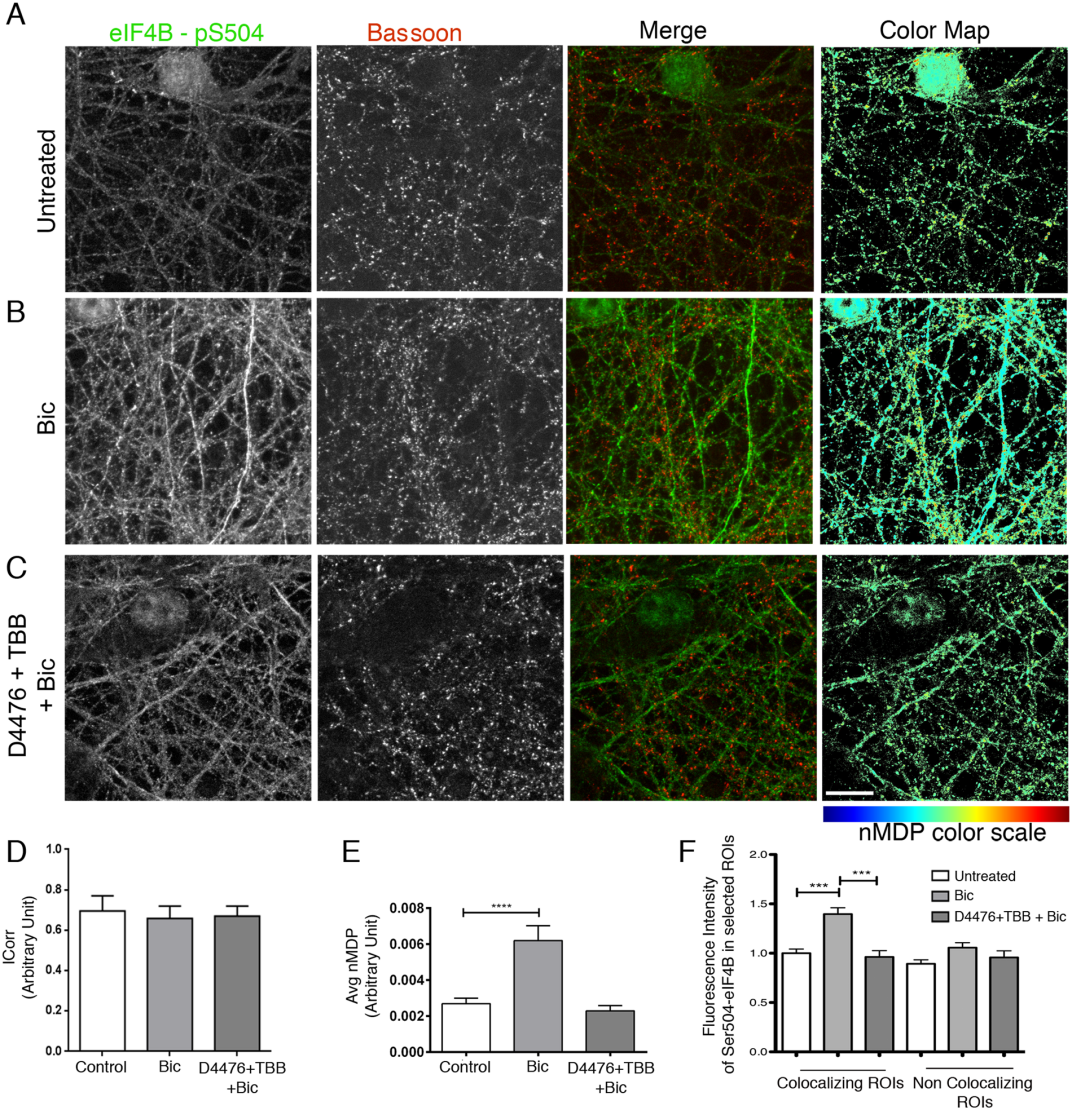
Effect of Ser504 phosphorylation on the subcellular localization of eIF4B in neurons. (**A**–**C**) Immunofluorescence with antibodies against Ser504-phosphorylated eIF4B (green) and the synaptic marker Bassoon (red) in neurons at rest (Untreated) or upon 15-min stimulation with 30 μM bicuculline (Bic) or pre-treated for 2 h with CK inhibitors (10 μM D4476 + 25 μM TBB) before bicuculline stimulation (D4476 + TBB + Bic). In bicuculline-treated neurons, Ser504 phosphorylation is clearly increased and this increase is prevented by CK inhibitors treatment. (**D**,**E**) Quantification of (**A**–**C**) images shows that the ICorr score is similar in all conditions (**D**) while the average nMDP score reveals that colocalization between Ser504 phosphorylated eIF4B and Bassoon is increased after bicuculline but not in the presence of CK inhibitors (**E**). Data are expressed as mean values ± SEM; n = 30 pictures from three independent experiments. Statistical significance is determined by one-way ANOVA followed by Dunnett post hoc test; ****P < 0.0001. (**F**) Fluorescence intensity of the Ser504-phosphorylated eIF4B signal measured in co-localizing and not co-localizing ROIs, selected on the color maps above. The increase in Ser504 phosphorylation levels upon bicuculline treatment is detectable only in co-localizing ROIs and is abolished by pre-treatment with CK inhibitors. Data are expressed as mean values ± SEM; n = 1350 ROIs for each experimental condition on images obtained from three independent experiments. Statistical significance is calculated using one-way ANOVA followed by Dunnett post hoc test; ***P < 0.001. Bar in (**A**–**C**): 10 μm.

Since we had observed that elevation of spontaneous synaptic activity was accompanied by an increase in eIF4B phosphorylation at Ser504, we investigated whether co-localization with Bassoon was also influenced by the neuronal activity. As expected, bicuculline stimulation promoted an increase in phospo-Ser504 labeling that was prevented by CK inhibitors (D4476 and TBB – Fig. 5A–C). The ICorr value was similar in all conditions, indicating that the co-localization between Ser504-phosphorylated eIF4B and Bassoon was not qualitatively altered by neuronal activity (Fig. 5D). However, when co-localization was quantified in terms of nMDP values, Ser504-phosphorylated eIF4B appeared increased in Bassoon-positive synaptic areas, an effect that was reverted by treatment with CK inhibitors (Fig. 5E,F). Overall, a picture emerges in which Ser540-phosphorylated eIF4B is enriched in synaptic microdomains and this preferential localization is increased by synaptic activity.

### Role of Ser504 phosphorylation in the recruitment of eIF4B to the translation pre-initiation complex

**Figure 6:**
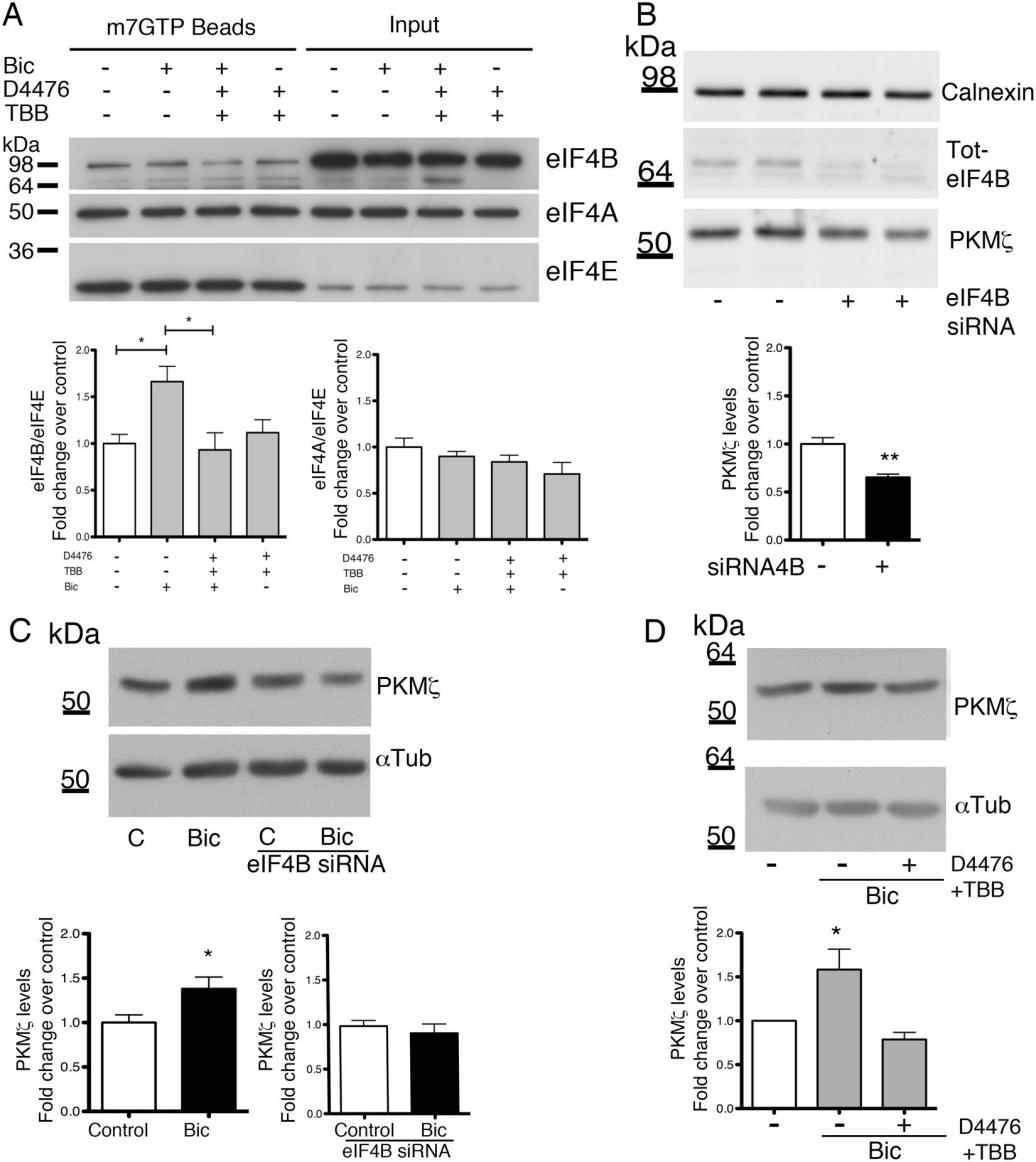
Influence of Ser504 phosphorylation on the association of eIF4B with the eIF4F complex. (**A**) Representative western blots (upper panel) and quantification (lower panel) of eIF4B, eIF4A and eIF4E in primary hippocampal neurons pre-treated for 2 h with two CK inhibitors (10 μM D4476 and 25 μM TBB) and exposed for 30 min to 30 μM bicuculline (Bic) before co-precipitation with 7-methyl-GTP-conjugated Sepharose beads. eIF4B and eIF4A levels are normalized against eIF4E levels and shown as fold change over control (untreated neurons). (**B**) Expression of eIF4B (Tot-eIF4B) and PKMζ in total cell extracts of hippocampal neurons transfected with eIF4B siRNA pool. (**C**) Expression of PKMζ in total cell extracts of hippocampal neurons treated with 10 μM bicuculline for 1 hour (Bic), either transfected (left panel) or not (right panel) with eIF4B siRNA pool (eIF4B siRNA). (**D**) Expression of PKMζ, in total cell extracts of hippocampal neurons pre-treated for 2 h with 10 μM D4476 and 25 μM TBB and then analyzed at rest or after treatment with 10 μM bicuculline (Bic) for 1 hour. Data are shown as mean ± SEM of at least three independent experiments (A, n = 4; B, n = 6; C, n = 6; D, n = 5). Statistical significance was calculated using the one-way ANOVA followed by Dunnett post hoc test (A, D) or unpaired two-tailed Student’s t-test in B-C (*P < 0.05; **P < 0.01).

It is known that eIF4B phosphorylation promotes its association with the translation pre-initiation complex^4^ and that Ser422 phosphorylation is important for the interaction with eIF3^17^. We tested whether phosphorylation of Ser504 was also able to influence the recruitment of eIF4B to the pre-initiation complex by co-precipitation with 7-methyl-GTP-coated Sepharose beads to mimic the 5′cap. In order to amplify neuronal activity, a condition known to increase translation initiation in neurons^47^, neuronal cultures were exposed to bicuculline in either the absence or the presence of the two CK inhibitors that proved effective on Ser504 phosphorylation. Western blot analysis of the proteins co-precipitated with the beads showed that pretreatment with D4476 and TBB reduced eIF4B recruitment to the initiation complex promoted by bicuculline. The effect was specific for eIF4B, since eIF4A recruitment was not influenced by either bicuculline or the kinase inhibitors (Fig. 6A). These findings indicate that Ser504 phosphorylation can modulate the translation initiation efficiency in neurons by favoring the association of eIF4B with the 5′-cap binding initiation complex eIF4F. In light of the proposed function of eIF4B in supporting the helicase activity of eIF4A, this effect can be particularly relevant for the translatability of mRNAs with highly structured transcript leaders.

### Ser504 eIF4B phosphorylation affects the translation of PKMζ

In light of the results described above, we investigated whether Ser504 phosphorylation regulates eIF4B activity in the translation process. We focused our attention on the translational control of PKMζ in cortical neurons based on the following considerations: (i) its mRNA is characterized by a structured 5′UTR; (ii) its translation is reported to be regulated by eIF4B phosphorylation^45^; and (iii) it is rapidly synthesized under conditions of increased synaptic activity leading to LTP induction^48^. The knock down of endogenous eIF4B by siRNAs significantly reduced PKMζ expression (Fig. 6B). Conversely, when the neuronal activity of the network was elevated by administration of bicuculline, which increases eIF4B Ser504 phosphorylation (see Fig. 2A), the protein levels of endogenous PKMζ significantly increased (Fig. 6C). This effect was prevented when bicuculline was applied to neurons in which the endogenous expression of eIF4B was knocked down (Fig. 6C). In addition, pre-treatment of neurons with CK inhibitors (D4476 and TBB), which specifically reduce Ser504 phosphorylation (see Fig. 2A and B), prevented the bicuculline-induced up-regulation of PKMζ (Fig. 6D).

Since PKMζ translation was reported to be increased by dephosphorylation of Ser406^45^, we also tested the phosphorylation state of this residue upon bicuculline treatment and CK inhibition. Under these conditions, the phosphorylation levels of Ser406 were not significantly changed (Fig. S5A and B), suggesting that the modulation in PKMζ levels primarily depends on Ser504 phosphorylation.

This body of results demonstrates that an increased synaptic activity elevates the level of eIF4B Ser504 phosphorylation, which, in turn, promotes the translation of PKMζ. Moreover, in light of the features of the PKMζ transcript leader, we can hypothesize that this mechanism of translation regulation applies to other neuronal mRNAs with structured 5′UTR.

### eIF4B phosphorylation at the Ser504 site is increased in an animal model of epilepsy

Finally, we evaluated whether phosphorylation of Ser504 takes place *in vivo* and whether this event is modulated by neuronal activity in an animal model of epilepsy, a disease characterized by pathological hyper-excitability that has already been used to probe the activity-dependency of eIF4B phosphorylation states^45^.

**Figure 7:**
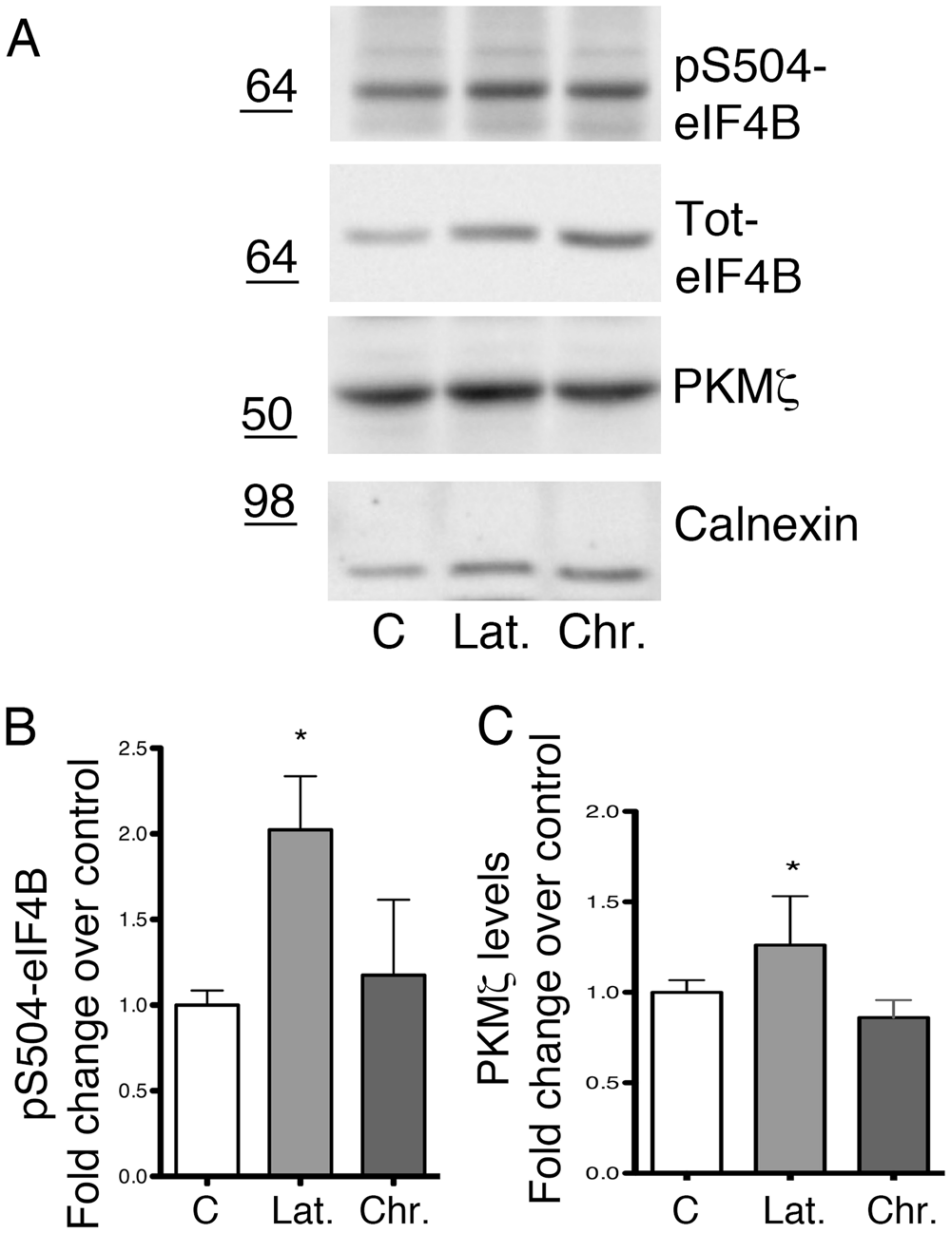
Ser504 phosphorylation of eIF4B in an animal model of epilepsy. Representative western blot and quantification of phospho-Ser504 eIF4B (pS504-eIF4B) and PKMζ levels in extracts prepared with RIPA buffer from adult rat cortex. Control animals (**C**) are compared with animals that underwent the epilepsy induction protocol, sacrificed either in the latency period (Lat.) or 50 days after occurrence of the first spontaneous seizure (Chr.). Phosphorylated eIF4B levels are normalized against total eIF4B and for loading, and shown as fold change over control (control animals). PKMζ levels are normalized for loading and shown as fold change over control (control neurons). Data are expressed as mean values ± SEM; n = 6 animal for each group. Statistical significance is determined with a nonparametric Kruskal-Wallis one-way analysis of variance followed by Dunns post test; *P < 0.05.

Human epilepsy can develop months or years after a brain lesion caused by a trauma, a stroke, or an episode of prolonged seizures (status epilepticus, SE). We used a rat model in which SE induced by a drug (pilocarpine) is followed, after a latency period of about 10 days, by spontaneous recurrent seizures^49^. Animals were sacrificed 6 days after SE (latency period) or 50 days after the occurrence of the first spontaneous seizure (chronic epilepsy period). Phosphorylation of Ser504 was significantly increased during latency, i.e. when plastic changes are occurring that lead to brain transformation from normal to epileptic^50^, but not in the chronic period (Fig. 7A,B). Interestingly, the high levels of eIF4B phosphorylation at the Ser504 site were paralleled by an increased translation of PKMζ (Fig. 7A,C). These data suggest that Ser504 phosphorylation may underlie plastic changes not only *in vitro* but also *in vivo*.

## Discussion

It is widely recognized that eIF4B phosphorylation, which increases its association with the pre-initiation ribosomal complex^4, 16, 51^, modulates protein translation^19^ and influences cell survival and proliferation in mitotic cells. In this work, we describe a neuron-specific regulation of eIF4B phosphorylation at the previously uncharacterized Ser504 site. Our *in vitro* results show constitutive phosphorylation of Ser504 in neurons, which is further increased under conditions of synaptic activation induced by bicuculline. We found that also Ser422 phosphorylation, which has been extensively studied in highly proliferating cells^15–18^, is controlled by neuronal activity, being decreased by TTx and increased by bicuculline. However, Ser422 phosphorylation is influenced by the activity of the ionotropic glutamate receptors with the contribution of VOCCs, whereas Ser504 phosphorylation appears to be mainly sustained by the tonic activity of the metabotropic glutamate receptors. This differential involvement of glutamate receptors corresponds to the engagement of distinct signaling pathways.

We found that, analogously to what observed in proliferating cells^4, 17^, the PI3K/mTOR and the MAPK pathways participate in Ser422 phosphorylation in neurons, with the first mainly regulating basal phosphorylation levels and the latter intervening in conditions of increased synaptic activity. The interplay between the MAPK and the PI3K/mTOR pathways has already been recognized to be important in hippocampal neurons for the regulation of protein synthesis, by differentially affecting the phosphorylation of various components of the translation machinery in response to neuronal activity^47, 52^. Interestingly, these two pathways do not contribute to Ser504 regulation, further highlighting the independent control of phosphorylation at the two sites.

We demonstrate that activation of Ser504 phosphorylation is primarily sustained by the constitutive activation of CKs, and is further boosted by cPKCs. While cPKCs, well-know players in neuronal physiology, are expected to exert pleiotropic roles in the phosphorylation of eIF4B, CKs appears to selectively act on the Ser504 site, although the relationship, if any, between cPKCs and CKs remains to be understood. In our experimental conditions, CKs appear to be responsible for the high levels of basal phosphorylation of Ser504 and to contribute to the modulation seen upon mGluR activation. Indeed, CKs, in particular CK1, are characterized by a high constitutive activity that can be further modulated by mGluRs in neurons^53, 54^. In addition, CK-dependent eIF4B phosphorylation has been reported in both rabbit reticulocytes and plants, even though the phosphorylation sites were unknown^55, 56^. Recent reports suggest a relevant role of CKs in translation regulation, achieved through inhibition of the eukaryotic translation initiation factor 4E-binding protein 1 (4E-BP1)^57^, the coordination of the ternary complex formation and eIF4F assembly^58^, and the modulation of the interaction between eIF4G, eIF4A and eIF4B in the helicase complex^59^. Here we describe a novel mechanism through which CKs positively modulate the translation initiation of mRNAs: by mediating Ser504 phosphorylation, CKs promote eIF4B recruitment to the initiation complex. In addition, we show that CK-mediated Ser504 phosphorylation modifies eIF4B subcellular localization upon increased neuronal activity, leading to its accumulation at *bona fide* synaptic sites. This observation points to the possibility that Ser504-phosphorylated eIF4B may participate in the regulation of local translation according to the levels of synaptic activity. Of note, this role can be exerted by modulating not only the interaction of the translation machinery with the mRNA, but also the helicase activity of the eIF4F complex. This latter effect is expected to be particularly relevant for transcripts endowed with structured transcript leaders, which are abundant in the pool of synaptic and stress regulated proteins^45^. This is the case of PKMζ, a protein with a highly structured transcript, which has been proposed to be involved in the induction and maintenance of long-term synaptic changes. We provide experimental evidence that eIF4B Ser504 phosphorylation exerts a translational control on the expression of endogenous PKMζ. Therefore, our data support a relevant contribution of eIF4B Ser504 phosphorylation on PKMζ expression, in addition to the role recently proposed for Ser406 phosphorylation in this process^45^. Further studies are needed in order to identify and characterize the full panel of neuronal transcripts whose translation is specifically regulated by eIF4B.

To verify whether Ser504 phosphorylation is regulated by neuronal activity also *in vivo*, we decided to employ a rat model of epilepsy, in order to compare the situation of normal brain activity with that of a hyper-excitable system. Interestingly, while Ser504 phosphorylation levels are unchanged in animals in the chronic phase (i.e. in condition of overt epilepsy), they are increased in the latency period, when the epileptic phenotype is not apparent yet, but maladaptive plastic changes are ongoing and eventually lead to spontaneous seizures. These changes in phosphorylation are expected to reflect an alteration of the neuronal activity since it is highly unlikely that they may be ascribed to the presence of a different population of cells. Indeed, if a certain extent of white blood cell infiltration is reported in animal models of epileptogenesis, this is observed immediately after the status epilepticus, while in the late latency phase is minimal; in any extent, even considering their possible contribution, white cells may represent only a tiny percentage of total cells, compared to neurons. Moreover, we did not detect Ser504 phosphorylation in blood-rich tissues such as liver, heart and kidney.

The epileptogenic process is characterized by profound alterations in neural circuitries, including specific changes in synaptic transmission and strength that depend on the molecular composition of synaptic contacts and on local protein synthesis^60^. Great attention has been paid in the last years to the understanding of the – still elusive – molecular mechanisms underlying epileptogenesis, in the continuous search for new therapeutic targets able to modify disease progression, given that the available therapies for epilepsy are only symptomatic^61^. Our findings prompt the provocative hypothesis that modulation of eIF4B phosphorylation (including, but possibly not exclusively at the Ser504 site) may play a role in the molecular mechanisms of epileptogenesis. This hypothesis, when confirmed by further investigation, may lead to the identification of new therapeutic targets for the prevention of epilepsy development.

In conclusion, we show that eIF4B, through its tightly regulated phosphorylation, appears as a key player in the control of protein translation in neurons. eIF4B is a point of convergence of multiple signaling pathways and may represent a crucial “fine tuner” in the local control of mRNA translation, when active protein synthesis is required. This may be relevant not only in physiological conditions, but also under pathological conditions that depend on stable changes in synaptic transmission and neuronal network activity.

## Materials and Methods

### Materials

Cell culture media and reagents were from Lonza (Basel, Switzerland), culture flasks and multiwell plates from Nalge Nunc (Rochester, NY, USA) and Petri dishes from Falcon BD (Franklin Lakes, NJ, USA). DL-2-amino-5-phosphonopentanoic acid (APV), 7-nitro-2,3-dioxo-1,4-dihydroquinoxaline-6-carbonitrile (CNQX), (RS)-1-aminoindan-1,5-dicarboxylic acid (AIDA), tetrodotoxin (TTx), bicuculline, NNC55-0396, (±)-BAYK8644 were from Tocris (Bristol, UK). Gö6976 and U0126 were from Merck Millipore (Billerica, MA, USA). Chymostatin, Leupeptin, Antipain, Pepstatin, Nimodipine, Rapamycin, LY294002, 1,2-dioctanoylglycerol (DiC8), 4-(4-(2,3-Dihydrobenzo[1,4]dioxin-6-yl)-5-pyridin-2-yl-1H-imidazol-2-yl)benzamide (D4476), 4,5,6,7-Tetrabromo-2-azabenzimidazole, 4,5,6,7-Tetrabromobenzotriazole (TBB) and other chemicals for general use were from Sigma-Aldrich (St Louis, MO, USA).

Antibodies against eIF4B (total and Ser422 phosphorylated forms), eIF4E, eIF4A, and ribosomal protein S6 were from Cell Signaling Technology (Danvers, MA, USA). Rabbit monoclonal antibody against phospho-S504 eIF4B was from Abcam (Cambridge, UK). Other mouse monoclonal antibodies were against Bassoon (Synaptic Systems GmbH, Goettingen, Germany), alpha-tubulin (Sigma-Aldrich), PSD95 (UC Davis/NIH NeuroMab Facility, Cat# 73-028, RRID:AB_10698024, Antibodies Inc., Davis, CA, USA for immunofluorescence and Abcam for Western blotting). Anti eIF4B C-terminal polyclonal antiserum was raised by Eurogentec (Seraing, Belgium), immunizing rabbits against the rat C-terminal sequence of the protein (SVDGEDEDEGDDCTE).

### Expression plasmids

Human eIF4B sequence was obtained from pcDNA3-Flag4Bwt (kind gift of J. Hershey). Ser504 mutants were generated by PCR: products obtained with overlapping primers containing the mutation were used as template for a subsequent PCR with external primers and inserted into pcDNA3-Flag. Oligonucleotides used (mutations in lower case) were: 5′-GAG CAG CAG gCC CCT ACA AGT GGT-3′ (forward) and 5′-ACC ACT TGT AGG GGc CTG CTG CTC-3′ (reverse) for eIF4B S504A; 5′-CAG AGC AGC AGg agC CTA CAA GTG GTG G-3′ (forward) and 5′-CCA CCA CTT GTA GGc tcC TGC TGC TCT G-3′ (reverse) for eIF4B S504E; 5′-CCG CTC GAG CTG CTT CTA TCT TTG GAG-3′ (forward) and Reverse: 5′-CCG CTC GAG CGG CCG CCT ATT CGG CA-3′ (reverse) as common external primers. All inserts were sequenced.

### Cell culture and treatments

The Institutional Animal Care and Use Committee approved the animal use. Hippocampal neurons were prepared according to ref. 62 from 2 to 3 day-old Sprague–Dawley rats. Briefly, the tissue was incubated into Hank’s solution containing 3.5 mg/ml trypsin type IX (Sigma-Aldrich) and 0.5 mg/ml DNase type IV (Calbiochem, La Jolla, CA, USA) for 5 min, and then mechanically dissociated in a Hank’s solution supplemented with 12 mM MgSO_4_ and 0.5 mg/ml DNase IV. Cells were plated onto poly-ornithine coated coverslips and maintained at 37 °C in a 5% CO_2_ incubator in minimal essential medium with 20 mM glucose, B27 (Life Technologies, Carlsbad, CA, USA), 2 mM glutamax, 5% fetal clone III (FCIII; Hyclone, South Logan, UT, USA) and 5 μM 1-β-D-cytosine-arabinofuranoside (Sigma-Aldrich). Neurons were used between 7 and 15 days after plating.

Cortical astrocytes were obtained from 1–2 day-old Sprague-Dawley rats (Charles River, Wilmington, MA, USA) according to ref. 63. Briefly, cortices were dissociated with 2.5 mg/ml trypsin type IX and 1 mg/ml DNase (10 min at 37 °C) in two steps without trituration. Cells were plated in 75 cm^2^ flasks with Minimum Essential Medium Eagle with Earle’s balanced salt solution, supplemented with 10% donor horse serum, 33 mM glucose, 2 mM glutamax (Gibco), 50 U/ml penicillin and 50 μg/ml streptomycin, and kept at 37 °C in a 5% CO_2_ incubator. Two steps of overnight shaking at 230 rpm were performed to induce selective detachment of microglia. After reaching confluence, astrocytes were re-plated onto poly-lysine-coated multiwells and used within 3 days.

HEK293 cells were maintained in Dulbecco’s modified Eagle’s medium supplemented with 10% FCIII, 2 mM glutamine, 10 mM Na pyruvate, 50 U/ml penicillin and 50 μg/ml streptomycin.

TTx 1 μM, bicuculline 10 μM, BAYK8644 20 μM, nimodipine 10 μM, NNC 55–0396 10 μM, and DiC8 100 μM were used for the indicated times. In some experiments cells were pre-treated before the bicucullin treatment as follows: (i) 100 μM APV, 20 μM CNQX and 1 mM AIDA, 30 min; (ii) 20 nM rapamycin, 10 mM U0126, or 25 μM LY294002, 2 h; (iii) Gö6976 2 μM, 1 h; (iv) D4476 10 μM and TBB 25 μM, 2 h.

### Western blotting

Cells were lysed with 2x SDS sample buffer (100 mM Tris–HCl, pH 6.8, 5 mM EDTA/Na, 4% SDS, 10% glycerol, 0.4 M DTT, 0.02% bromophenol blue). After SDS-PAGE proteins were blotted. After blocking, membranes were incubated overnight with the primary antibodies and, after washing, with horseradish peroxidase-conjugated secondary antibodies (Bio-Rad, Hercules, CA, USA). For loading controls membranes were stripped in acidic buffer (0.2 M glycine, 0.1% SDS, 1% Tween-20, pH 2.2) and re-probed. Proteins were revealed on auto-radiographic films (GE Healthcare, Piscataway, NJ, USA) by Super Signal West Chemiluminescent Substrate (ThermoFisher Scientific, Waltham, MA, USA) and quantified using ImageJ^64^.

### Biochemical procedures

#### Tissue homogenization

Tissues were homogenized in radio-immunoprecipitation assay (RIPA) buffer (50 mM Tris-HCl pH 8.0, 150 mM NaCl, 0.5 mM EDTA/NaOH, 1% Triton X-100, 1% Na-deoxycholate, CLAP, 1 mM NaF, 2 mM Na_3_VO_4_) with 30 strokes of a glass-Teflon homogenizer and centrifuged at 15,000 g, 4 °C for 15 min. The protein content was analyzed by BCA (ThermoFisher Scientific).

#### Synaptosomal preparation

Synaptosomes from the cortex of adult rats were prepared as described previously^65^. The cortex from 1 rat was homogenized in 2 ml of homogenization solution (0.32 M sucrose, 0.1 mM CaCl_2_, 1 mM MgCl_2_, CLAP) at 4 °C using a Teflon-glass homogenizer, brought to 1.5 M sucrose and divided into two ultracentrifuge tubes (SW41Ti 14 × 89mm). The homogenate was overlaid with 3 ml of 1.25 M sucrose, 3 ml of 1 M sucrose and 3 ml of homogenization solution, and then centrifuged at 100,000 g for 4 h at 4 °C. Synaptosomes were collected at the 1.25 M/1.0 M sucrose interface and centrifuged, after 1:4 dilution with phosphate buffered saline solution (PBS) at 100 000 g for 1 h at 4 °C. The pellet was then solubilized (1% Triton X-100, 10 mM Tris/HCl pH7.5, 60 mM NaCl, 1 mM MgCl_2_, 0.1 mM CaCl_2_, CLAP) before SDS-PAGE analysis on 7.5% SDS gels and transfer of proteins to nitrocellulose membranes for Western blotting analysis.

#### Two-dimensional gel electrophoresis

Cells were lysed with two-dimensional electrophoresis buffer (8 M urea, 10 mM Tris-HCl pH 7.5, 2% CHAPS, 18 mM DTT, 1% IPG pH 3–10NL buffer ampholine, CLAP, 1 mM NaF, 2 mM Na_3_VO_4_) and extracts were applied to 18 cm IPG strips pH 3–10 non-linear (GE Healthcare) for isoelectrofocusing (IPGphor system, GE Healthcare). Second dimension was then performed on 7.5% SDS-PAGE gels and proteins transferred onto nitrocellulose membranes for Western blotting analysis.

#### λ-phosphatase (λPPase) treatment

Neurons were lysed in lysis buffer (10 mM Tris-HCl pH 7.6, 2% CHAPS, 1% NP-40, CLAP). 50 μl of 20 mM MnCl_2_ solution, 50 μl of 10x λ-PPase buffer (50 mM Hepes/NaOH pH 7.5, 100 mM NaCl, 2 mM DTT, 0.01% Brij-35) and bi-distilled water were added to 400 μg of protein to a final volume of 500 μl. The mixture was divided into two aliquots and 600 units of λPPase (New England Biolabs, Hitchin, United Kingdom) were added to one of the aliquots. Aliquots were incubated for 6 h at 30 °C and proteins were acetone-precipitated at −20 °C before SDS-PAGE. Dephosphorylation of HEK293 cell lysate before immunoprecipitation was performed in the same manner, except that the lysate was incubated for 2 h at 30 °C.

#### Immunoprecipitation

Transfected HEK293 cells were lysed in lysis buffer (0.05 M Tris/HCl pH 7.5, 0.2% NP-40, 150 mM NaCl, 50 mM β-glycerol phosphate, 1 mM NaF, 2 mM EDTA, 1.5 mM MgCl_2_, 10% glycerol, CLAP). Lysates were treated with λPPase and then immunoprecipitated with Protein A-sheparose (Sigma-Aldrich) conjugated with anti-Flag polyclonal antibody (Sigma-Aldrich) at 4 °C overnight. After three washes with lysis buffer, proteins were eluted with 2x SDS sample buffer and analyzed by Western blotting.

#### 5′mRNA-cap complex analysis

Proteins were extracted from neurons (7–9 days *in vitro*) with lysis buffer (10 mM potassium phosphate buffer pH 7.5, 1 mM EDTA/Na, 10 mM MgCl_2_, 50 mM β-glycerolphosphate, 5 mM EGTA/Na, 0.5% NP-40, 0.1% Brij-35, 0.1% sodium deoxycholate, CLAP, 1 mM NaF, 2 mM Na_3_VO_4_) and 400 μg were incubated with 7-methyl GTP Sepharose 4 B beads (GE Healthcare) in the presence of 100 μM GTP overnight at 4 °C. The beads were washed three times (lysis buffer without Brij-35, with 0.1% NP-40) and eluted with 2x SDS sample buffer.

### Cell transfection

HEK293 cells were plated the day before transfection at 50% of confluence in poly-L-Lysine (Sigma-Aldrich) treated 6 well plates and then transfected with 1.8 μg of DNA (1.5 μg of plasmid encoding WT-eIF4B and 300 ng of plasmid encoding GFP) and 4 μl of Lipofectamine 3000 (Life Technologies), according to the manufacturer’s instructions. After 24 h cells were lysed (phosphate buffered saline containing 10 mM EDTA/Na, 2% NP-40, 0.2% SDS, CLAP, 1 mM NaF, 2 mM Na_3_VO_4_), protein content determined with the BCA reagent and then used for immunoprecipitation or Western blotting.

Neurons (4 days *in vitro*) were transfected with 100 pmol of siRNA (ON-TARGETplus NON-TARGETING rat POOL and ON-TARGETplus SMART POOL against rat eIF4B - ThermoFisher Scientific) and 4 μl of Lipofectamine 3000 (Life Technologies), according to the manufacturer’s instructions. At 8 days *in vitro* neurons were collected, lysed in PBS (containing 10 mM EDTA/Na, 1% TX-100 and protease inhibitor cocktail), total protein content determined with the BCA reagent (ThermoFisher Scientific, Pierce) and then analysed by Western blotting.

### Immunofluorescence

Neurons were grown on glass coverslips for 8 days *in vitro*, fixed with 4% paraformaldehyde solution, containing 120 mM phosphate buffer pH 7.4 and 4% sucrose and incubated at room temperature for 2 h with primary antibodies, followed by 1 h with fluorescent secondary antibodies (Jackson Immunoresearch Laboratories, West Grove, PA, USA). Antibodies were diluted in goat serum dilution buffer, containing 15% goat serum (Gibco-Life Technologies), 450 mM NaCl, 0.1% Triton X-100, 20 mM phosphate buffer at pH 7.4, as in ref. 66. Coverslips were mounted in Dako Fluorescent Mounting Medium (Dako Italia, Milano, Italy). Dephosphorylation was accomplished by alkaline phosphatase treatment of fixed cells, performed for 2 h at 37 °C (30 U/ml alkaline phosphatase in 50 mM Tris buffer, 0.1 mM EDTA pH 8), according to ref. 67.

Images were acquired with a laser scanning confocal microscope (TCS SP2 or SP8 SMD FLIM, Leica microsystem, Manheim, Germany) with a 63x/1.4 NA objective (2x or 4x zoom, 1024 × 1024 pixels). Z-stacks were collected at 20 μm intervals and maximum projections were obtained using the Leica confocal software.

### Image Analysis

Image analysis was performed with ImageJ program^64^. Co-localization analysis was performed using the ImageJ Colocalization Colormap Plugin, based on ref. 46. This plugin provides a correlation of intensity values between pairs of individual pixels in two different channels calculating the normalized mean deviation product (nMDP) for each pixel. Results are presented as a Color map (hot colors for positive and cold colors for negative correlation). The plugin calculates the index of correlation (ICorr), which represents the fraction of positively correlated pixels in the image, the average positive nMDP value, the average nMDP value and the nMDP histogram of data.

The Color map was also used to select regions of interest (ROIs) in which the Ser504-eIF4B antibody signal was co-localized (red in the Color map image, red-ROIs) or not co-localized (green in the Color map image, green-ROIs) with the Bassoon signal. A total of 675 red- and 675 green-ROIs were selected for each experimental condition on images obtained from three independent experiments. Normalized fluorescence intensity of the Ser504-eIF4B signal was calculated for each ROI after subtraction of the mean fluorescence of background. Normalized fluorescence intensity values were finally analyzed with Prism software.

### Animals

Male Sprague-Dawley rats (250–350 g; Harlan, Italy) were used for *in vivo* experiments. All procedures and animal care were carried out in accordance with European Community, national and local approved guidelines, laws and policies. All protocols were approved by the University of Ferrara Ethical Committee for Animal Experimentation and by the Italian Ministry of Health (authorization number: D.M. 246/2012-B). Experiments were run following the ARRIVE guidelines^68^ and the NC3Rs recommendations^69^. Rats were housed under constant temperature (22–24 °C) and humidity (55–65%), 12 h light/dark cycle, free access to food and water. All animals were (i) acclimatized to laboratory conditions for 1 h before the start of the experiment, (ii) used only once during the pilocarpine protocol and (iii) euthanized at different times points by anesthetic overdose.

All experimental procedures were as described in ref. 70. In brief, rats received a single injection of methyl-scopolamine (1 mg/kg, s.c.) 30 min prior to pilocarpine (370 mg/kg, i.p.) or vehicle (0.9% NaCl solution; control animals). After pilocarpine injection, ~80% of rats developed a convulsive status epilepticus (SE) that was interrupted 3 h after onset by administration of diazepam (20 mg/kg, i.p.). They then entered a period of apparent wellbeing (latency phase) that ended 10 ± 1 days after SE with the first spontaneous seizures. Thereafter, rats remained in a chronic stage in which they regularly displayed spontaneous recurrent seizures. Test and time-matched control animals were killed at 2 different time points: 6 ± 1 days after SE (latency), or 50 days after the first spontaneous seizure (chronic). Brains were dissected and frozen for further analysis. 24/7 video monitor recordings were used to detect the presence (or to verify the absence in the latency phase) of convulsive seizures.

### Statistical analysis

Statistical analysis was performed with Prism software version 5.0 (GraphPad Software Inc., La Jolla, CA, USA). For biochemical experiments, columns in the graphs represent the mean (±SEM) of at least three independent experiments. For immunofluorescence analyses, columns in the graphs represent the mean (±SEM), obtained by a total of 10 z-stacks per experimental conditions, that were collected, processed and analyzed from three independent experiments. Statistical significance was evaluated (with 95% confidence intervals) by one-way ANOVA followed by Dunnett post hoc test (for multiple comparisons against a single reference group), Bonferroni post hoc test (for multiple comparisons between groups), two-tailed Student t-test (for comparisons between two average values), the nonparametric Mann-Whitney U test for significant differences between experimental groups in the co-localization analysis or the nonparametric Kruskal-Wallis one-way analysis of variance followed by Dunns post test for the analysis of the brain samples from the *in vivo* experiments. A value of P < 0.05 was considered to be statistically significant.

## Electronic supplementary material

Supplementary info
