# Supplementary material for: eIF4B phosphorylation at Ser504 links synaptic activity with protein translation in physiology and pathology
Source: Sci Rep. 2017 Sep 5;7:10563. doi: 10.1038/s41598-017-11096-1 (PMC5585320; doi:10.1038/s41598-017-11096-1)
Supplement: Supplementary file 1 — Supplementary info [file 41598_2017_11096_MOESM1_ESM.pdf]

eIF4B phosphorylation at Ser504 links synaptic activity with protein translation in physiology and pathology

Barbara Bettegazzi[1], Serena Bellani[1], Paolo Roncon[2], Fabrizia Claudia Guarnieri[1], Alice Bertero[1], Franca Codazzi[1,2], Flavia Valtorta[1,2], Michele Simonato[2,3], Fabio Grohovaz[1,2,*], Daniele Zacchetti[1,*]

[1]Unit of Cellular Neurophysiology and Unit of Neuropsychopharmacology, Division of Neuroscience, IRCCS San Raffaele Scientific Institute, via Olgettina 60, I-20132 Milano, Italy

[2]Vita-Salute San Raffaele University, via Olgettina 58, I-20132 Milano, Italy

[3]Department of Medical Sciences, University of Ferrara, via Fossato di Mortara 17-19, 44121 Ferrara, Italy

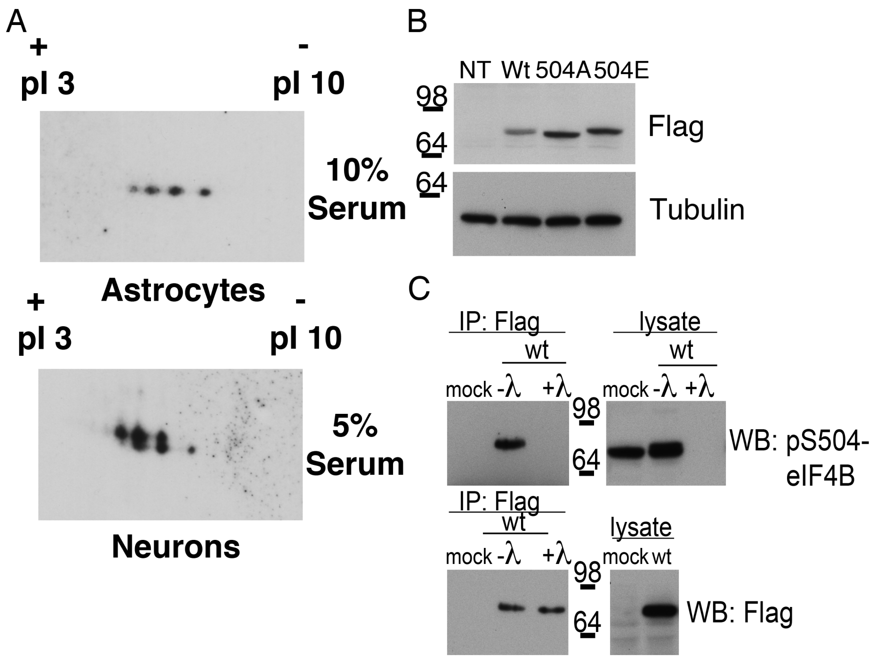

Figure S1 (**A**) Two-dimensional gel electrophoresis followed by western blot analysis of protein extracts of cortical astrocytes (upper panel) or neurons (lower panel) using the anti-N-terminal eIF4B antibody. Protein extracts are resolved by isoelectric point (pI) in the first dimension on a pH 3-10 non-linear range and by relative molecular mass in the second dimension by 7.5% SDS-PAGE. (**B**) Expression of eIF4B in HEK293 cells transfected for 24 h with the Flag-tagged wild type eIF4B (Wt) or its mutant forms with substitution of Ser504 with either alanine (504A) or glutamate (504E). The phosphomimetic form (504E) shows a slower migration than the others. (**C**) Western blot of immunoprecipitated Flag-tagged eIF4B (IP Flag-eIF4B) from extracts of HEK293 cells transfected for 24 h with the Flag-tagged wild type eIF4B (Wt) and treated or not with λ-phosphatase (λ). Either the anti-phospho-Ser504 eIF4B (pS504-eIF4B) antibody or an anti-Flag antibody were used on IP Flag-eIF4B. The crude extract before immunoprecipitation (lysate) was employed as a control under the same conditions. The antibody shows high specificity for phosphorylated Ser504.

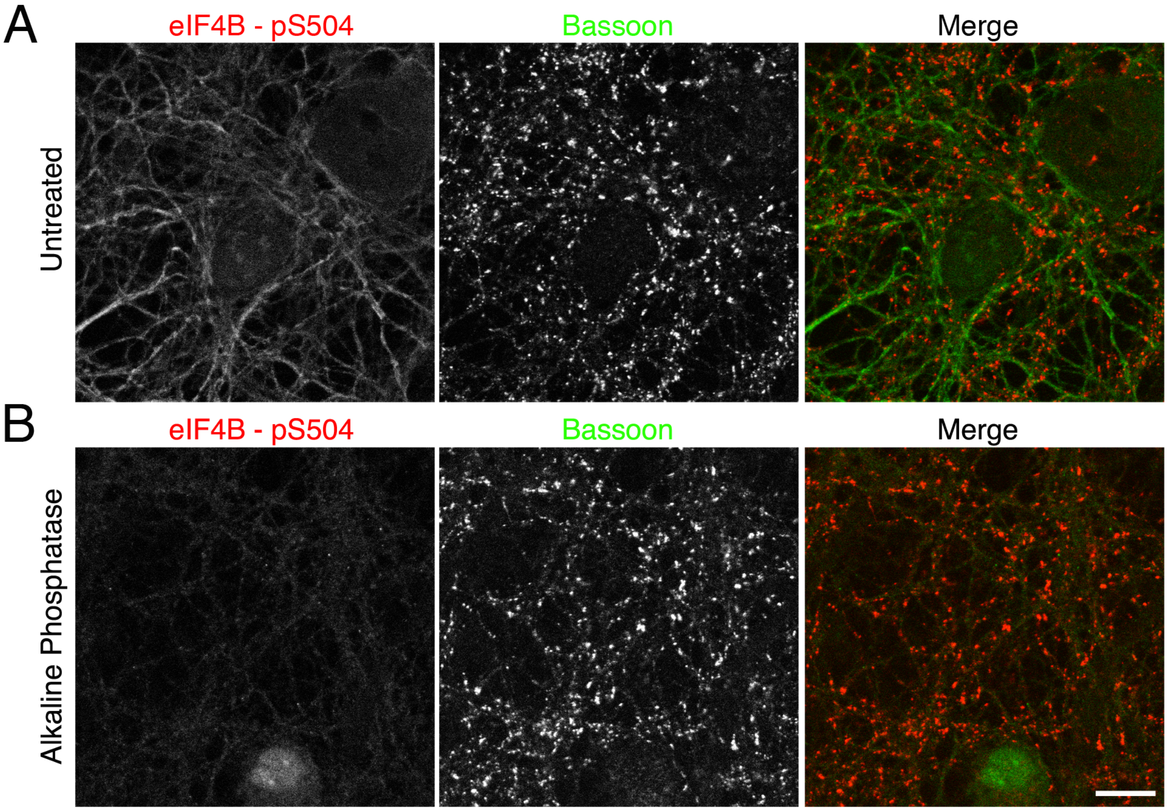

Figure S2 Specificity of the antibody against Ser504-phosphorylated eIF4B. Immunofluorescence analysis with antibodies against Ser504-phosphorylated eIF4B (green) and the synaptic marker Bassoon (red) in neurons either untreated (**A**) or treated with alkaline phosphatase (**B**). After treatment with alkaline phosphatase, the signal of the phospho-specific antibody is virtually abolished. Bar in (**A**, **B**): 10 μm.

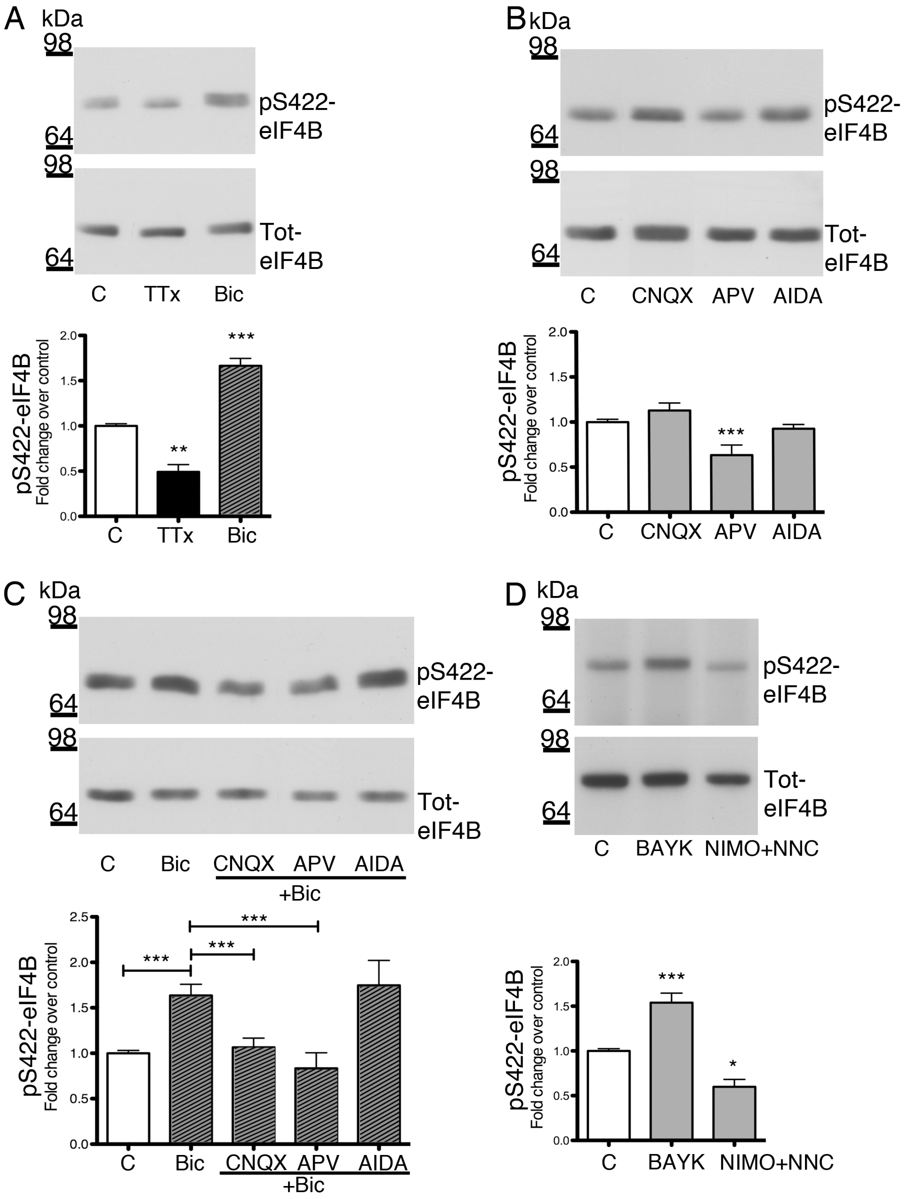

Figure S3 Regulation of eIF4B phosphorylation by neuronal activity. (**A-D**), Representative western blot (top) and quantification (bottom) of phospho-Ser422 eIF4B (pS422-eIF4B) levels in primary hippocampal neurons exposed to various drugs. (**A**) 1 μM TTx for 60 min (TTx) or 10 μM bicuculline for 30 min (Bic) (n=4 each group); (**B**) 30-min treatment with the following antagonists of GluRs: 100 μM APV for NMDA-Rs; 20 μM CNQX for AMPA-R/kainate-R; 1 mM AIDA for group I mGluRs (n=4); (**C**) 30-min treatment with 10 μM bicuculline after 30-min pre-incubation with GluR antagonists as in B (n=3 each group); (**D**) 10 μM BAYK8644 (60 min; BAYK) or 10 μM nimodipine + 10 μM NNC 55-0396 (60 min; Nimo+NNC) (n=3 each group). Phosphorylated eIF4B levels are normalized against total eIF4B and shown as fold change over control (untreated neurons). Data are shown as mean ± SEM of at least three independent experiments for each treatment. Statistical significance is calculated using one-way ANOVA followed by Bonferroni post hoc test; * $P<0.05$, ** $P<0.01$, *** $P<0.001$.

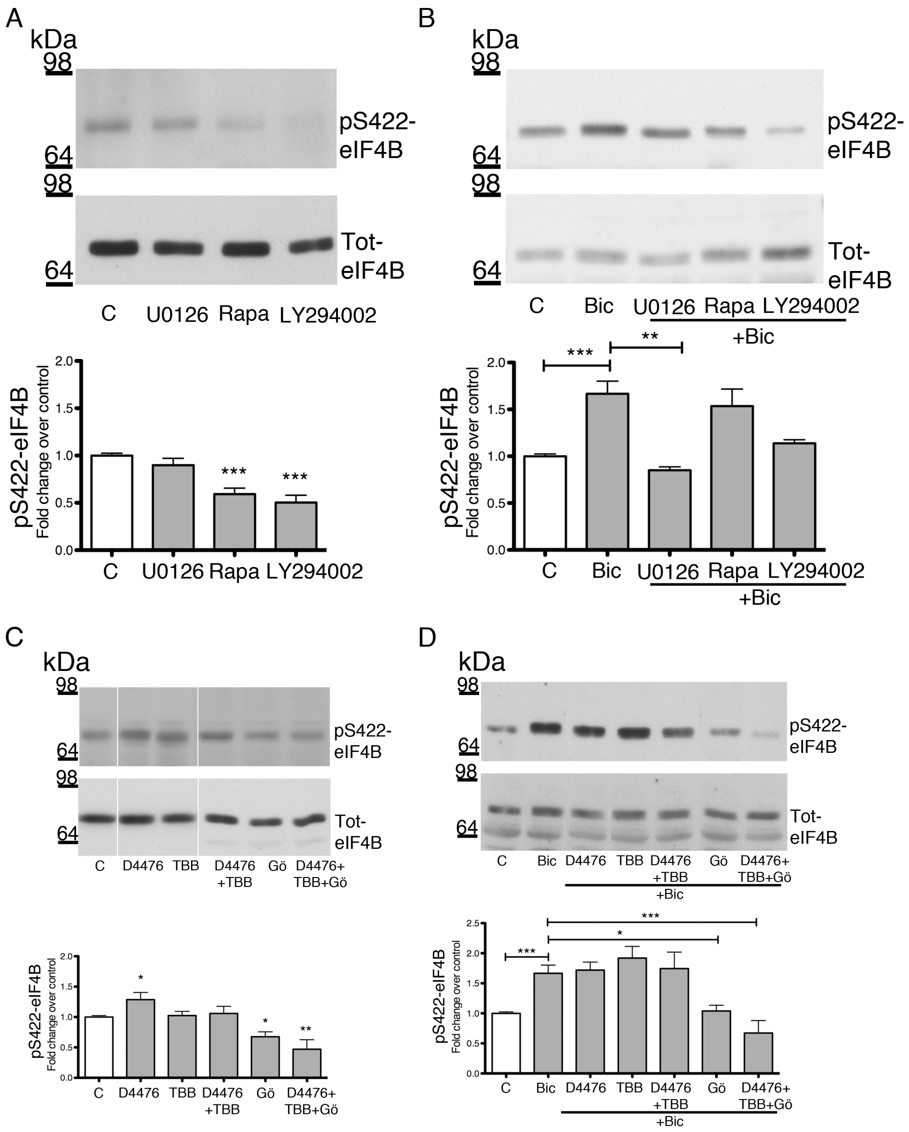

Figure S4 Involvement of protein kinases in the phosphorylation of eIF4B in neurons. (**A-D**), Representative western blot and quantification of phospho-Ser422 eIF4B (pS422-eIF4B) levels in primary hippocampal neurons treated for 3 h with 15 μM U0126, 20 nM rapamycin (Rapa), 25 μM LY294002 (**A, B***)* or pre-treated for 2 h with two CK inhibitors (10 μM D4476, 25 μM TBB) or a cPKC inhibitor (2 μM Gö6976; Gö), alone or in combination (**C, D***)* and analyzed at rest (**A, C***)* or after treatment with 10 μM bicuculline (Bic) for 30 min (**B, D***)*. Phosphorylated eIF4B levels are normalized against total eIF4B and shown as fold change over control (untreated neurons). Data are shown as mean ± SEM of at least three independent experiments for each treatment (**A**-**B**, n=3; **C**-**D**, n=4). Statistical significance is calculated using one-way ANOVA followed by Bonferroni post hoc test; * P<0.05, **P<0.01, *** P<0.001.

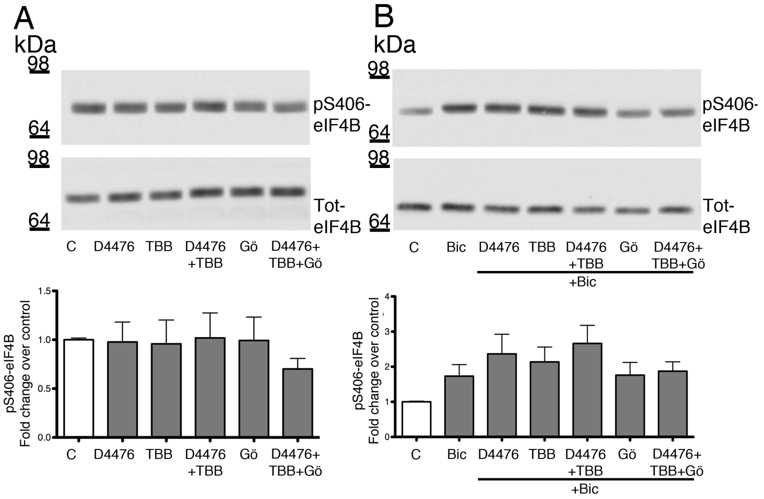

Figure S5 (**A-B**)**,** Representative western blot and quantification of phospho-Ser406 eIF4B (pS406-eIF4B) levels in primary hippocampal neurons pre-treated for 2 h with two CK inhibitors (10 μM D4476, 25 μM TBB) or a cPKC inhibitor (2 μM Gö6976; Gö), alone or in combination (**A, B**) and analyzed at rest (**A**) or after treatment with 10 μM bicuculline (Bic) for 30 min (**B**). Phosphorylated eIF4B levels are normalized against total eIF4B and shown as fold change over control (untreated neurons). Data are shown as mean ± SEM of at least three independent experiments for each treatment (**A**-**B**, n=3).

Original full-length Western blotting exposures used in the manuscript

1A Total eIF4B

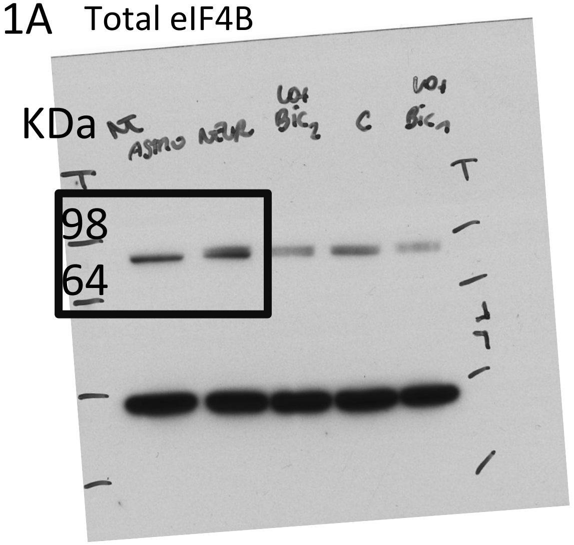

Total eIF4B

1B

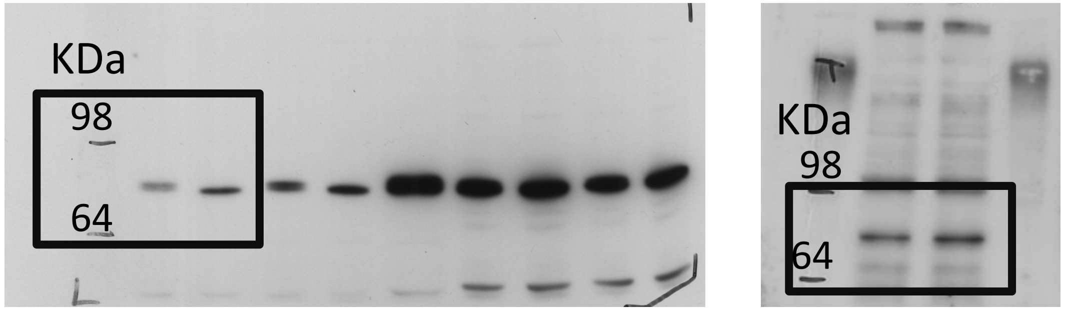

1C

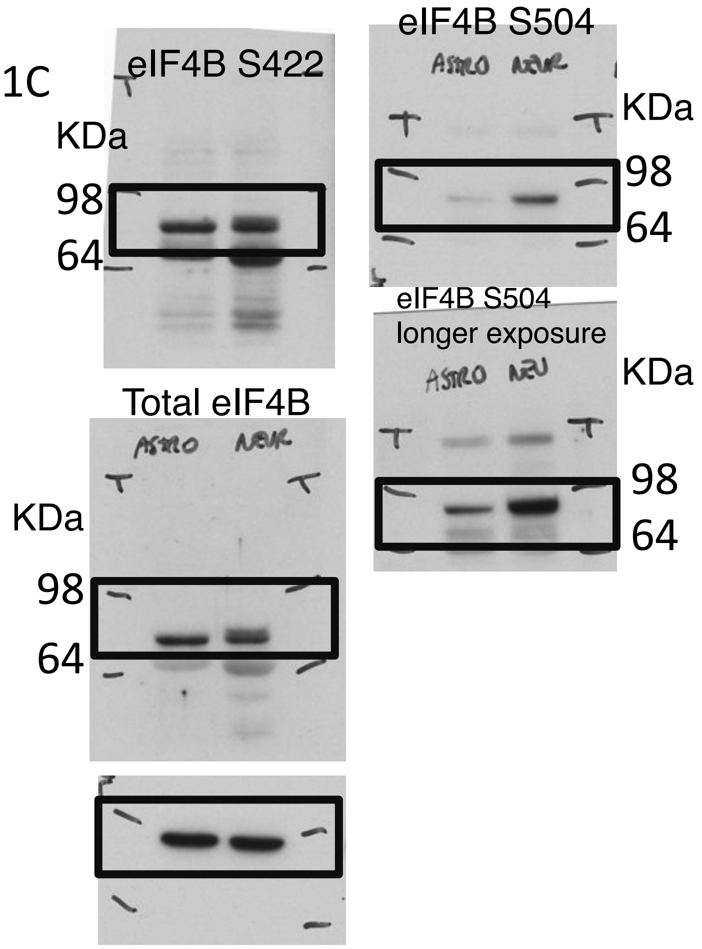
eIF4B S422
KDa
98
64
eIF4B S504
KDa
98
64
eIF4B S504
longer exposure
KDa
98
64
Total eIF4B
KDa
98
64

1D

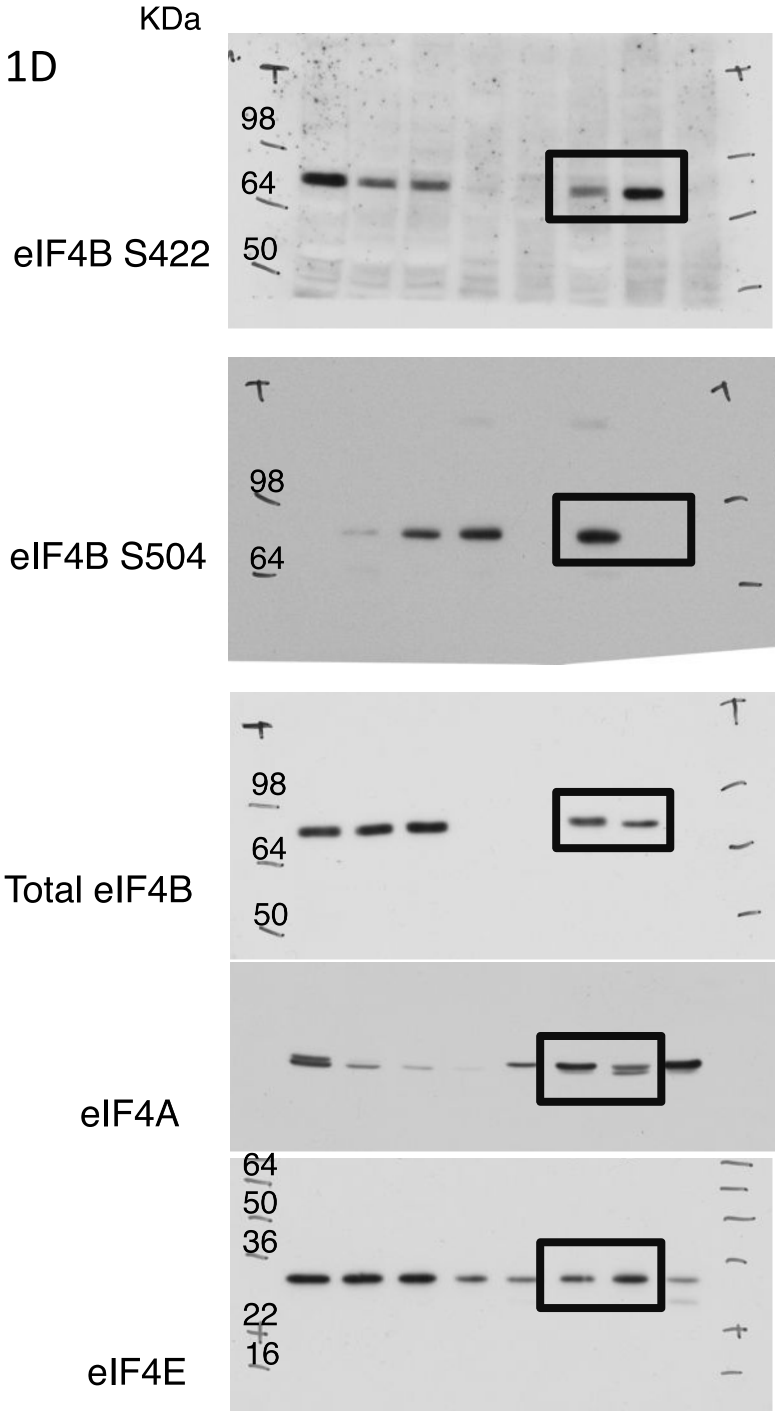
KDa
eIF4B S422
98
64
50
eIF4B S504
98
64
Total eIF4B
98
64
50
eIF4A
eIF4E
64
50
36
22
16

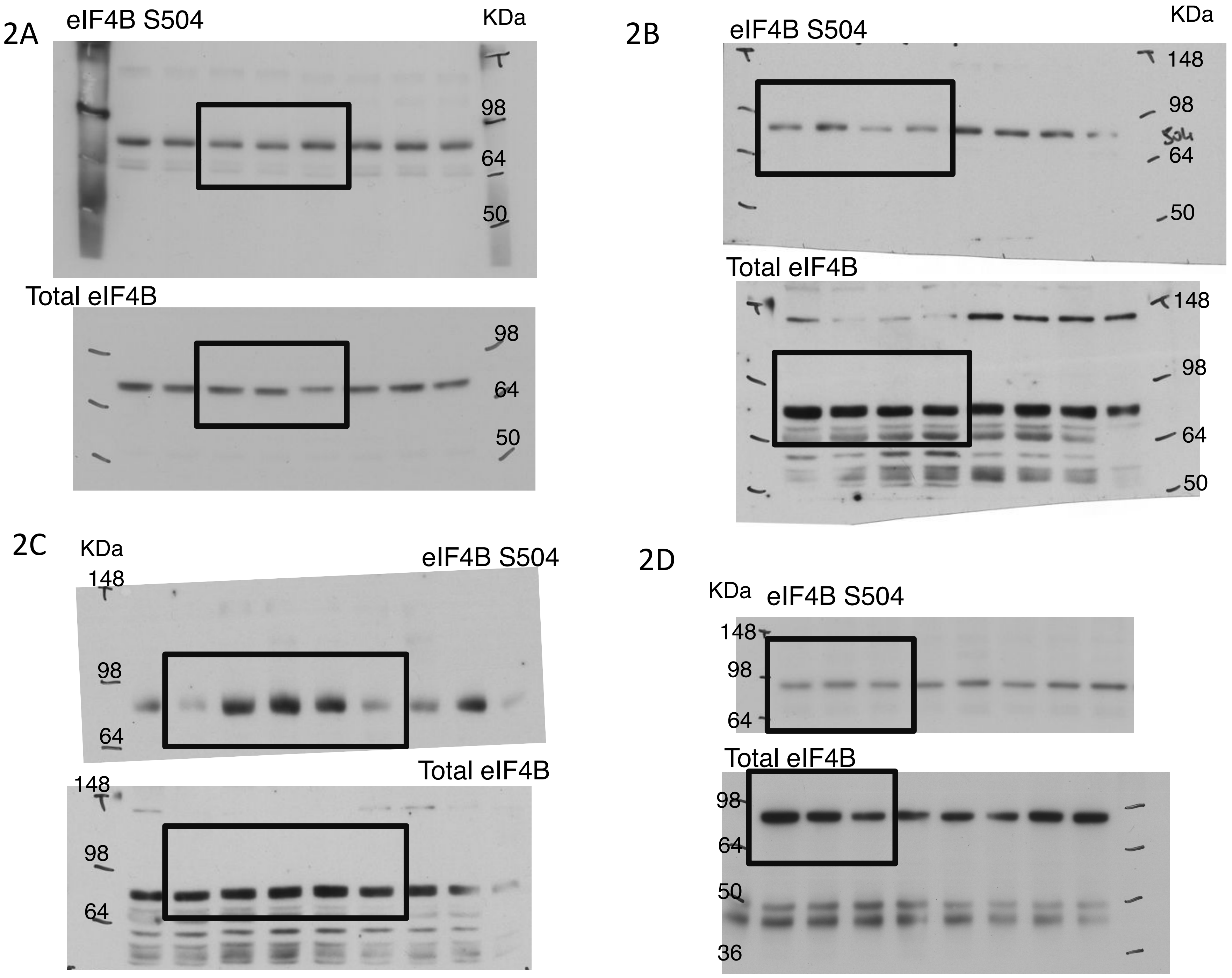

2A
eIF4B S504
KDa
98
64
50
Total eIF4B
98
64
50
2B
eIF4B S504
KDa
148
98
64
50
Total eIF4B
148
98
64
50
2C
KDa
eIF4B S504
148
98
64
Total eIF4B
148
98
64
2D
KDa
eIF4B S504
148
98
64
Total eIF4B
98
64
50
36

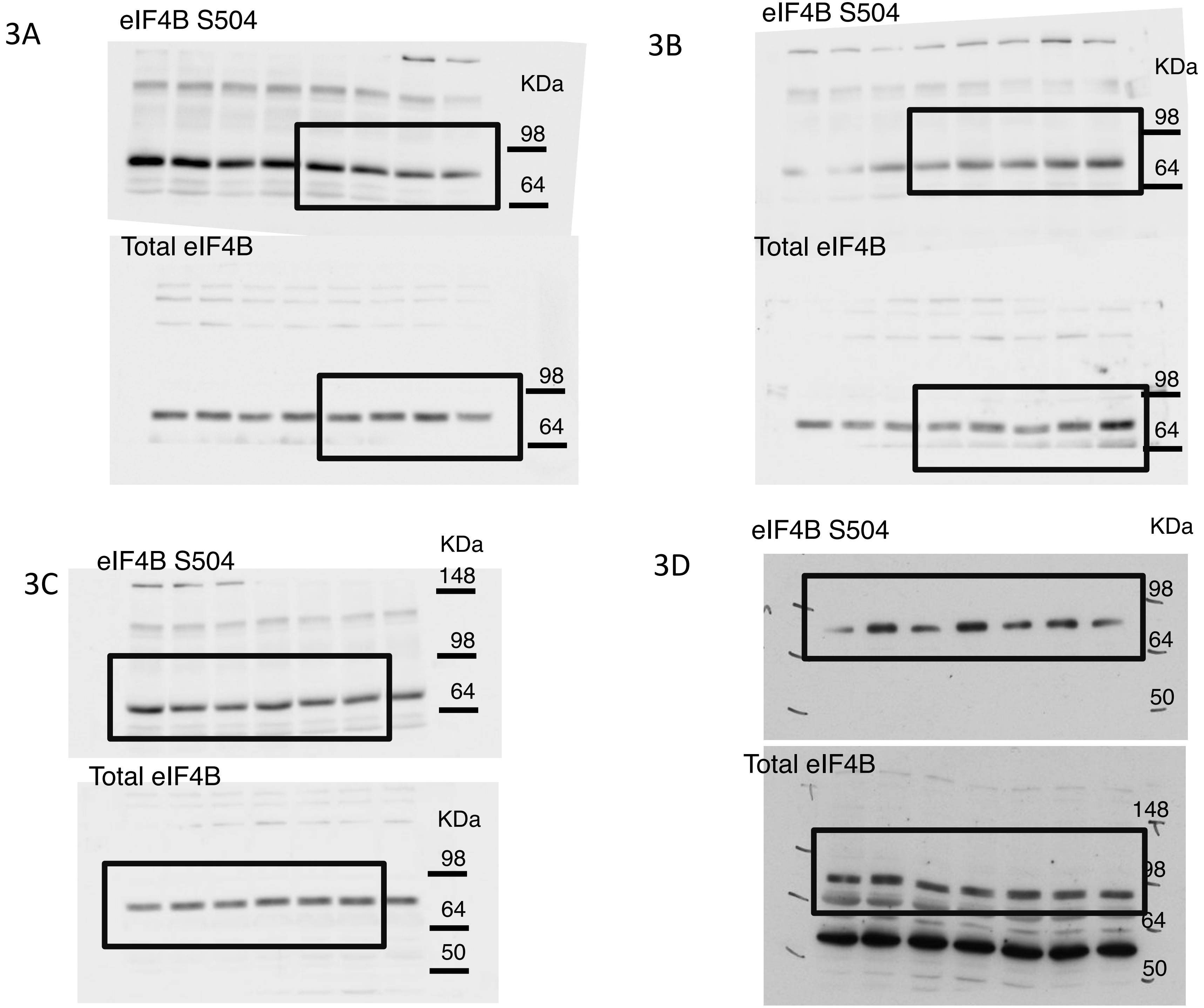
3A
eIF4B S504
KDa
98
64
Total eIF4B
98
64
3B
eIF4B S504
KDa
98
64
Total eIF4B
98
64
3C
eIF4B S504
KDa
148
98
64
Total eIF4B
KDa
98
64
50
3D
eIF4B S504
KDa
98
64
50
Total eIF4B
148
98
64
50

4I

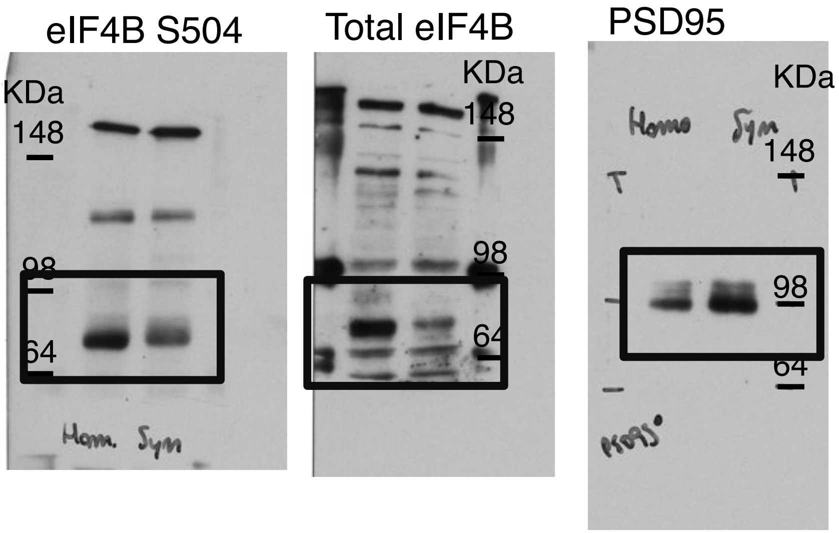
eIF4B S504
Total eIF4B
PSD95
KDa
148
98
64
KDa
148
98
64
KDa
148
98
64

6A

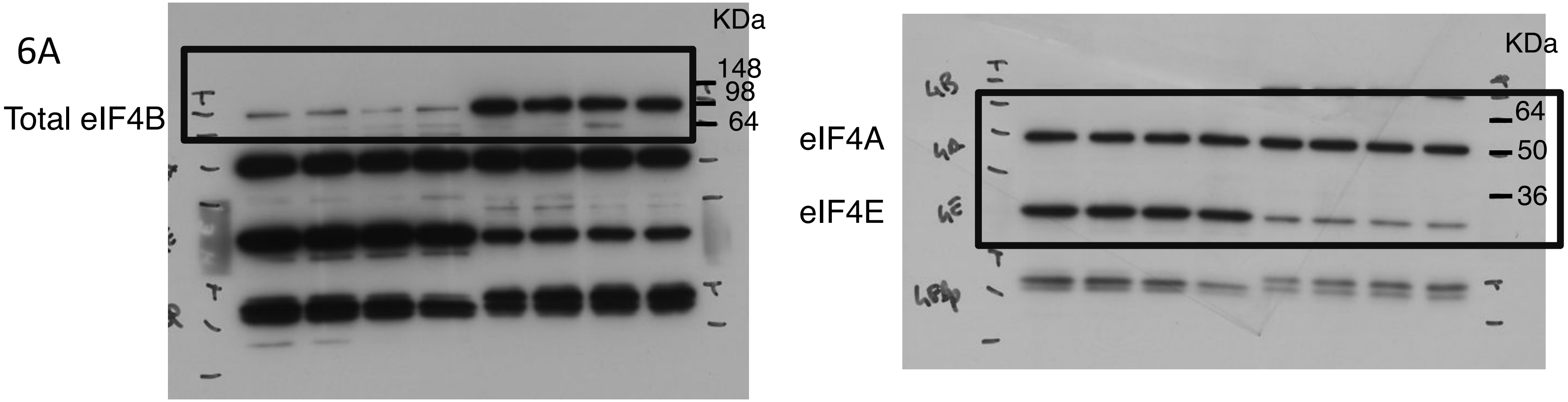
Total eIF4B
KDa
148
98
64
eIF4A
eIF4E
KDa
64
50
36

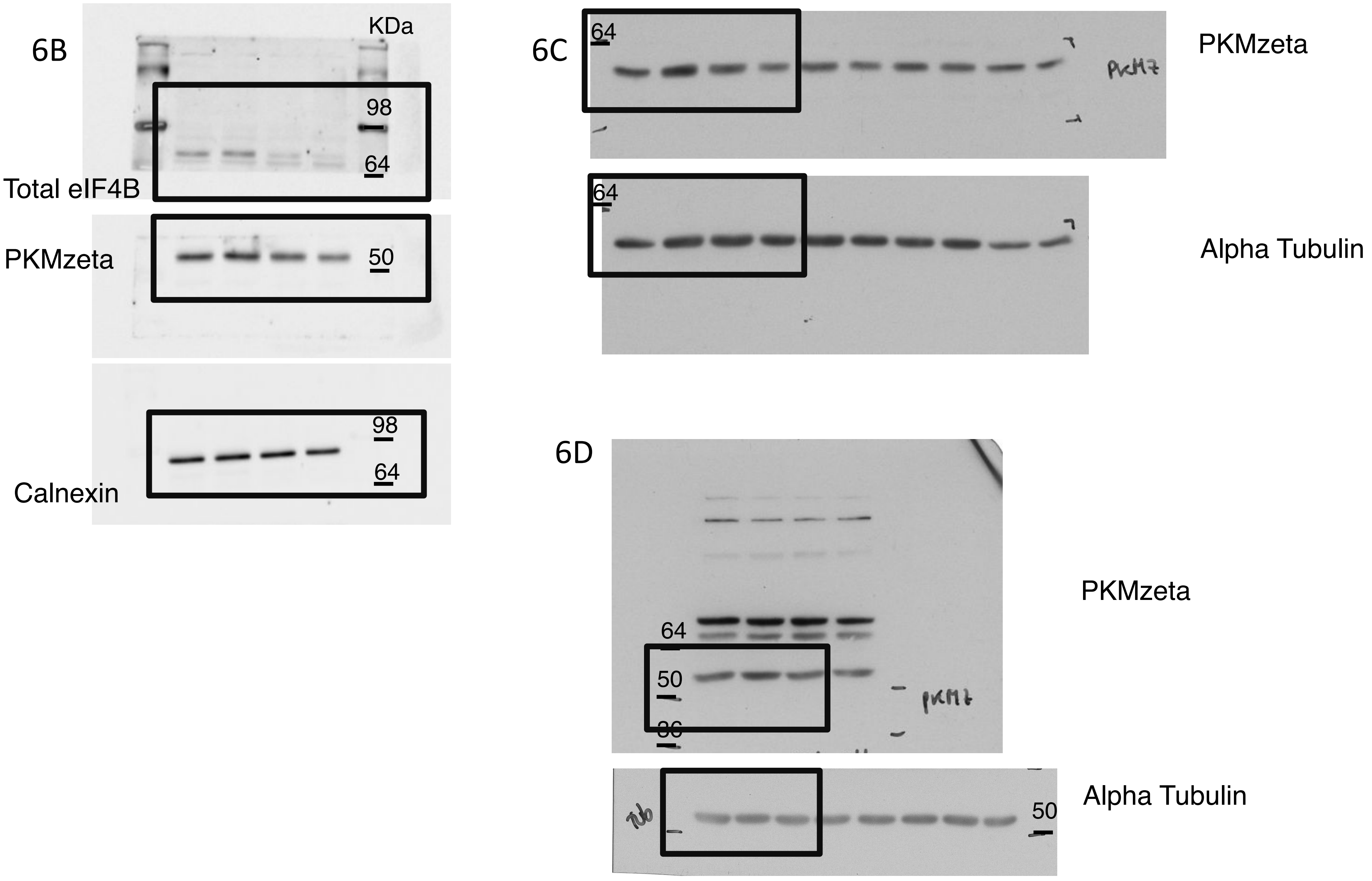
6B
KDa
98
64
Total eIF4B
PKMzeta
50
98
64
Calnexin
6C
64
PKMzeta
64
Alpha Tubulin
6D
PKMzeta
64
50
36
Alpha Tubulin
50

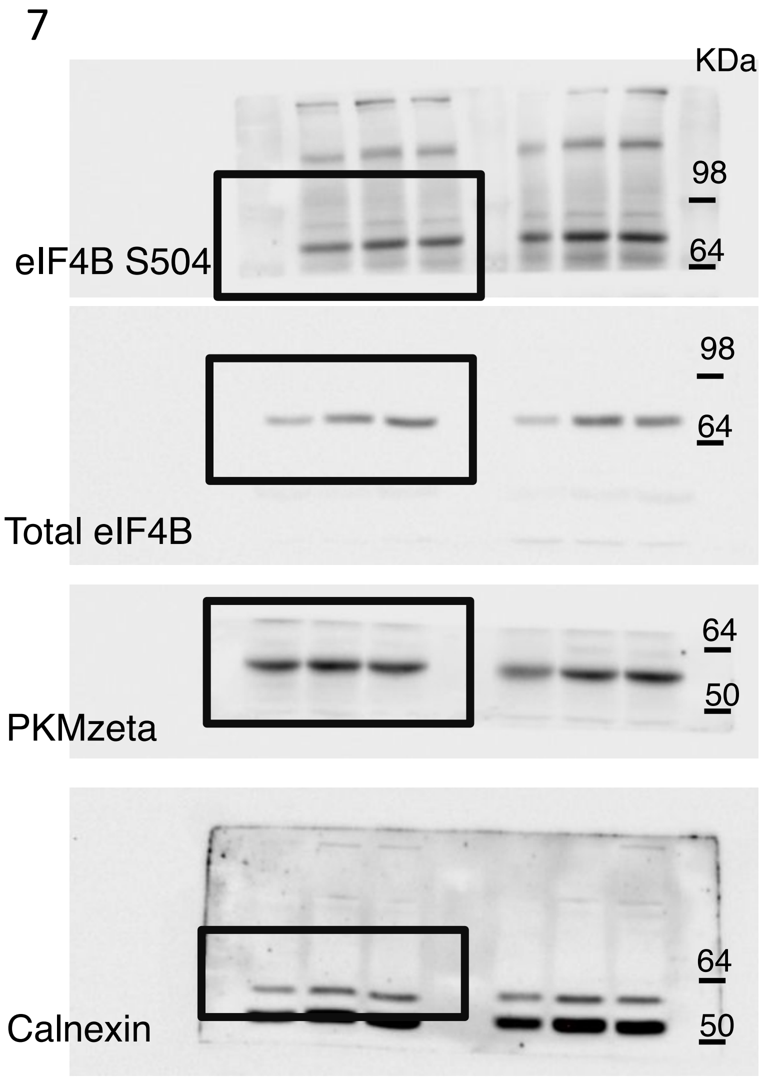
7
KDa
98
64
eIF4B S504
98
64
Total eIF4B
64
50
PKMzeta
64
50
Calnexin
